# Nanoscale Kevlar Liquid Crystal Aerogel Fibers

**DOI:** 10.1021/acsnano.2c06591

**Published:** 2022-09-02

**Authors:** Zengwei Liu, Jing Lyu, Yi Ding, Yaqian Bao, Zhizhi Sheng, Nan Shi, Xuetong Zhang

**Affiliations:** † School of Nano-Tech and Nano-Bionics, University of Science and Technology of China, Hefei 230026, P. R. China; ‡ Suzhou Institute of Nano-tech and Nano-bionics, Chinese Academy of Sciences, Suzhou 215123, P. R. China; § Division of Surgery and Interventional Science, University College London, London NW3 2PF, United Kingdom

**Keywords:** Kevlar nanofibers, liquid crystal, aerogel fibers, thermal insulation, information encryption

## Abstract

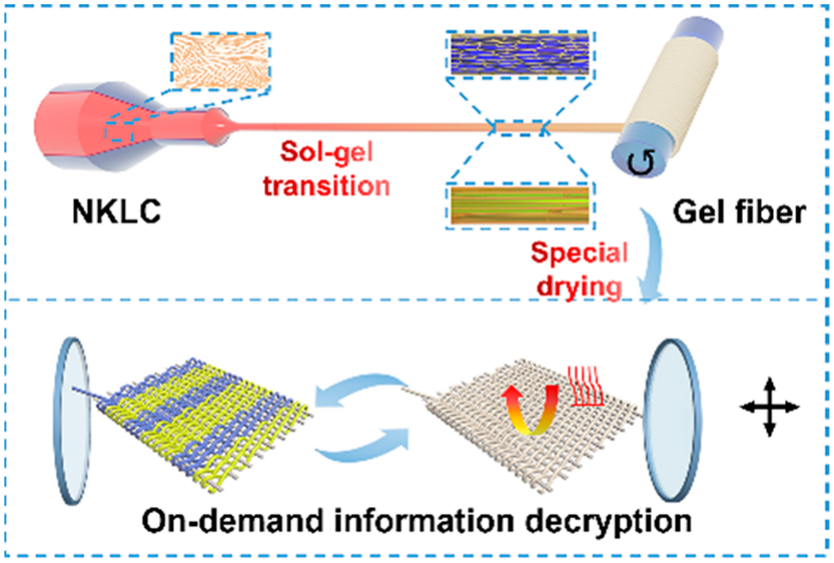

Aerogel fibers, the simultaneous embodiment of aerogel porous network and fiber slender geometry, have shown critical advantages over natural and synthetic fibers in thermal insulation. However, how to control the building block orientation degree of the resulting aerogel fibers during the dynamic sol–gel transition process to expand their functions for emerging applications is a great challenge. Herein, nanoscale Kevlar liquid crystal (NKLC) aerogel fibers with different building block orientation degrees have been fabricated from Kevlar nanofibers *via* liquid crystal spinning, dynamic sol–gel transition, freeze-drying, and cold plasma hydrophobilization in sequence. The resulting NKLC aerogel fibers demonstrate extremely high mechanical strength (41.0 MPa), excellent thermal insulation (0.037 W·m^–1^·K^–1^), and self-cleaning performance (with a water contact angle of 154°). The superhydrophobic NKLC aerogel fibers can cyclically transform between aerogel and gel states, while gel fibers involving different building block orientation degrees display distinguishable brightness under polarized light. Based on these performances, digital textiles woven or embroidered with high- and low-orientated NKLC aerogel fibers enable up to 6.0 Gb information encryption in one square meter and on-demand decryption. Therefore, it can be envisioned that the tuning of the building blocks’ orientation degree will be an appropriate strategy to endow performance to the liquid crystal aerogel fibers for potential applications beyond thermal insulation.

In recent years, the demand for lightweight, high-performance, and multifunctional fibrous materials has gradually increased due to their significant role in the field of composite engineering, textile engineering, environmental engineering, *etc*.^1−6^ Aerogel fibers, with the characteristics of ultralow density, ultrahigh porosity, and large specific surface area, have shown critical advantages over natural and synthetic fibers in thermal insulation, which have been regarded as the next generation high-performance thermal insulation fiber for human beings.^7−9^ In the past decade, tremendous efforts have been made to develop various aerogel fibers. For example, cellulose aerogel fibers have been fabricated for thermal encapsulation of diesel hybrid engines for fuel savings in cars.^10^ Silica aerogel fibers have been fabricated by reaction spinning, which exhibits excellent thermal insulation and wide temperature stability, showing great potential for wearable applications.^7^ Polyimide aerogel fibers have been fabricated *via* a sol–gel confined transition approach with low thermal conductivity (0.025–0.032 W·m^–1^·K^–1^) and a wide working temperature window (−165–250 °C), which might be applied as thermal insulation materials in harsh environments including cold protection and fire resistance.^11^ Additionally, a series of conductive aerogel fibers based on graphene sheets or metallic nanoparticles have been successfully fabricated, which have shown great potential in electronics, joule heating, and energy management.^12−14^ However, the functionality of these aerogel fibers mainly comes from their chemical components (such as the high electrical conductivity of graphene and the flame retardancy of polyimide), and there are few studies on the relationship between structure and performance other than thermal insulation properties. In previous work, the relationship between the particle size of the isotropic nanostructure units inside aerogel fibers and the light transmittance has been investigated.^7^ When the nanostructure units are anisotropic, whether their arrangement affects the properties of aerogel fibers remains elusive.

In fact, aerogel fibers are distinguished from aerogel monoliths, not only in geometry but also in the fabrication process. The traditional fabricating process of aerogels involves (1) a sol–gel transition process and (2) either a supercritical-drying process or a freeze-drying process. The sol–gel transition process for fabricating aerogel monoliths is static, which can be applied to make any variety of aerogels, even metal aerogels.^15^ However, to obtain aerogel fibers, reaction spinning^7^ or wet-spinning^8^ is usually applied, resulting in the dynamic sol–gel transition process. This dynamic process has stringent requirements on the size of the aerogel building blocks, where the molecular-scale building blocks are too small to accomplish the sol–gel process after being spun into the coagulation bath due to fast diffusion while the microscale building blocks are too large to obtain target aerogel fibers with a high specific surface area. Only nanoscale structures (*i*.*e*., nanofibers, nanoparticles, nanosheets, *etc*.) are the appropriate building blocks for constructing aerogel fibers.^7,11,16^ For example, Kevlar nanofibers^17−19^ as the nanoscale building block have been successfully utilized to fabricate aerogel fibers *via* the dynamic sol–gel transition, and the resulting Kevlar aerogel fibers with either fluorocarbon resin coating hydrophobilization^8^ or *in situ* small molecule hydrophobilization^20^ have shown excellent thermal insulation performance in comparison with the natural cotton fibers. However, the three-dimensional (3D) porous network constructed *via* Kevlar nanofibers in the resulting aerogel fibers was disordered and uncontrollable. Actually, it is another blank space to acquire aerogel fibers with ordered and controllable microstructure during the dynamic sol–gel transition process.

In this work, nanoscale Kevlar liquid crystal (NKLC) aerogel fibers were designed and fabricated to fill in the above-mentioned blank spaces. The nanoscale Kevlar was applied as the building blocks to form aerogel fibers with highly oriented structures due to its inherent advantages: (1) the high mechanical strength, (2) the liquid crystal states at high concentration, and (3) the high tolerance for drafting. Specifically, the NKLC was conducted to liquid crystal wet-spinning, dynamic sol–gel transition, freeze-drying, and cold plasma treatment in sequence to obtain the aerogel fibers with ordered and controllable microstructure. It should be mentioned that liquid crystal wet-spinning is an emerging and useful technology for fabricating fibrous materials from the liquid crystal spinning solution.^21−23^ For example, graphene aerogel fiber was fabricated by liquid crystal wet spinning.^12^ This NKLC aerogel fiber undoubtedly exhibits good thermal insulation (0.037 W·m^–1^·K^–1^) attributed to the aerogel structure. Importantly, these aerogel fibers with different building block orientation degrees will turn into corresponding gel fibers after soaking in an appropriate solvent (*e*.*g*., ethanol, and the resulting gel fibers exhibit different brightness in the field of polarized light, which can be utilized for information encryption and on-demand decryption. Thus, the NKLC aerogel fiber is a dual-functional fiber (*i*.*e*., both thermal insulation and information encryption), which would have broad prospects of application in the information era. Based on these, digital textiles with information (both bar code and two-dimensional code) encryption and on-demand decryption performance have been realized by hybrid weaving or embroidering these NKLC aerogel fibers with different building block orientation degrees. The resulting digital textiles hide information in the aerogel state and display it in the gel state under polarized light, exhibiting information encryption and on-demand decryption performance. Our work also gives inspiration for fabricating other aerogel fibers with controllable building block orientation, and the resulting aerogel fibers might have great potential in digital textiles for various wearable devices.

## Results and Discussion

**Scheme 1:**
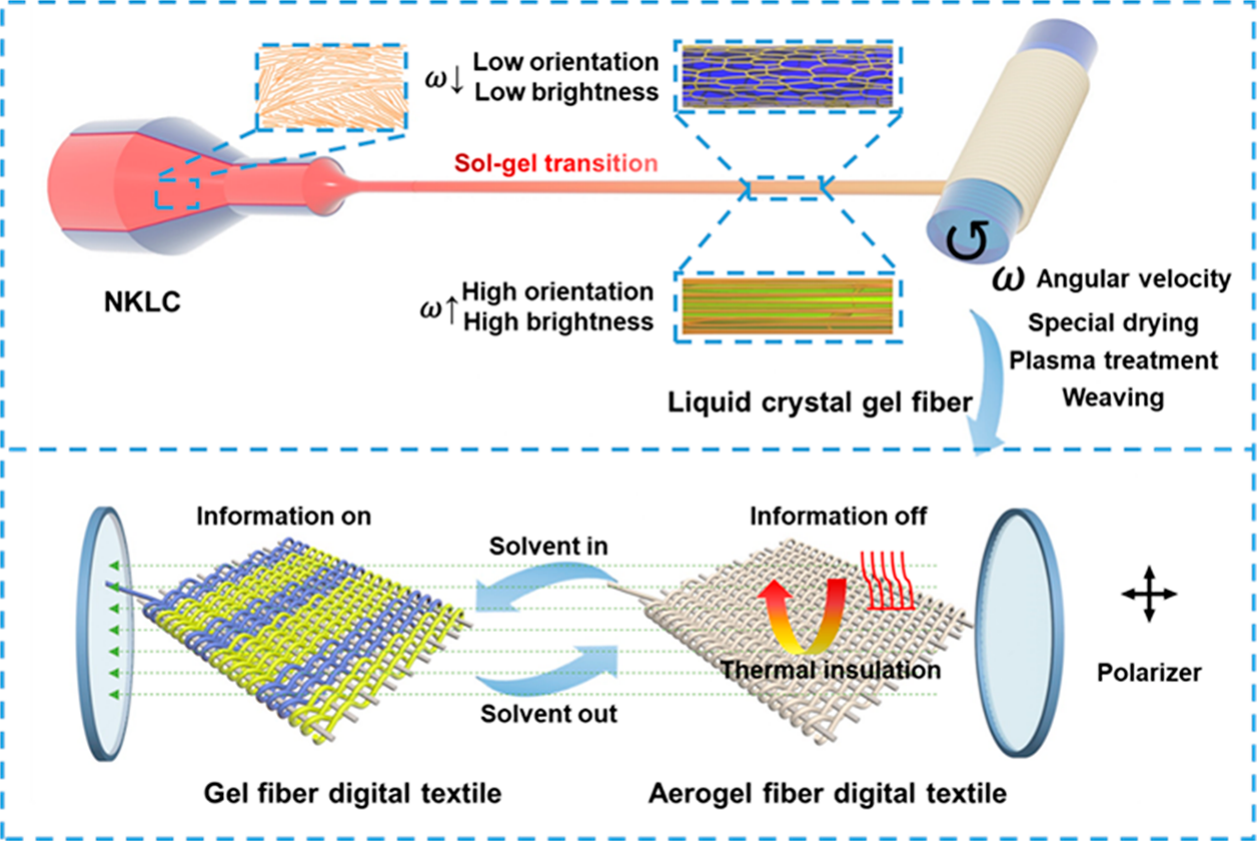
Schematic Fabrication Process Illustration of the Nanoscale Kevlar Liquid Crystal (NKLC) Aerogel Fibers and Their Application for Thermal Insulation, Information Encryption, and On-Demand Decryption

The fabrication process of the NKLC aerogel fibers is demonstrated in Scheme 1, which involves liquid crystal spinning, orientation controlling/sol–gel process, freeze-drying, and cold plasma treatment. Kevlar nanofibers were prepared by dissolving bulky Kevlar into dimethyl sulfoxide (DMSO) in the presence of methanol and potassium tert-butoxide, according to a report.^24−27^ The NKLC was formed *via* concentrating of the obtained Kevlar nanofiber dispersion, which is distinct from the traditional Kevlar liquid crystal. Because the NKLC is composed of nanofibers, which can be applied as nanoscale building blocks for the subsequent sol–gel transition, the traditional Kevlar liquid crystal is generated from rod-like poly(*p*-phenylene terephthalamide) macromolecules at a high concentration.^28^ The liquid crystal spinning was applied to extrude the NKLC into a coagulation bath (*i*.*e*., water). The ratio of the collection linear velocity to the extrusion linear velocity is defined as the draft ratio. The NKLC gel fibers with different building block orientation degrees were obtained by regulating the draft ratio during spinning. Although utilizing the draft ratio to control the orientation of an anisotropic building block is a traditional technique, which has not been applied to the fabrication of aerogel fibers, due to their generally poor mechanical properties. Since the birefringence index raises with the increase of orientation degree, the gel fibers with different building block orientation degrees will show distinguishable brightness and color under the same angle of polarized light. Furthermore, the solvent is removed by freeze-drying;^29,30^ thus, the liquid crystal aerogel fibers were obtained. The aerogel structure not only endows the high thermal insulation (0.037 W·m^–1^·K^–1^) but also preserves the building block orientation degree of the gel fibers. Cold plasma treatment is applied to obtain the superhydrophobic NKLC aerogel fibers, which could keep the oriented porous structure without shrinkage when exposed to moisture or immersed in water. Then, the NKLC aerogel fibers with two different building block orientation degrees were woven into a digital textile with the predetermined order for information encryption. To identify the predetermined pattern and decipher the information, two steps are required, *i*.*e*., transferring from aerogel textile into gel textile by absorbing ethanol and observing under polarized light. Attributing to the superhydrophobicity, the information encryption and decryption can be realized cyclically by simply ambient pressure drying and immersing in ethanol, respectively.

### Fabricating Process and Performance Test of NKLC Gel Fibers

**Figure 1:**
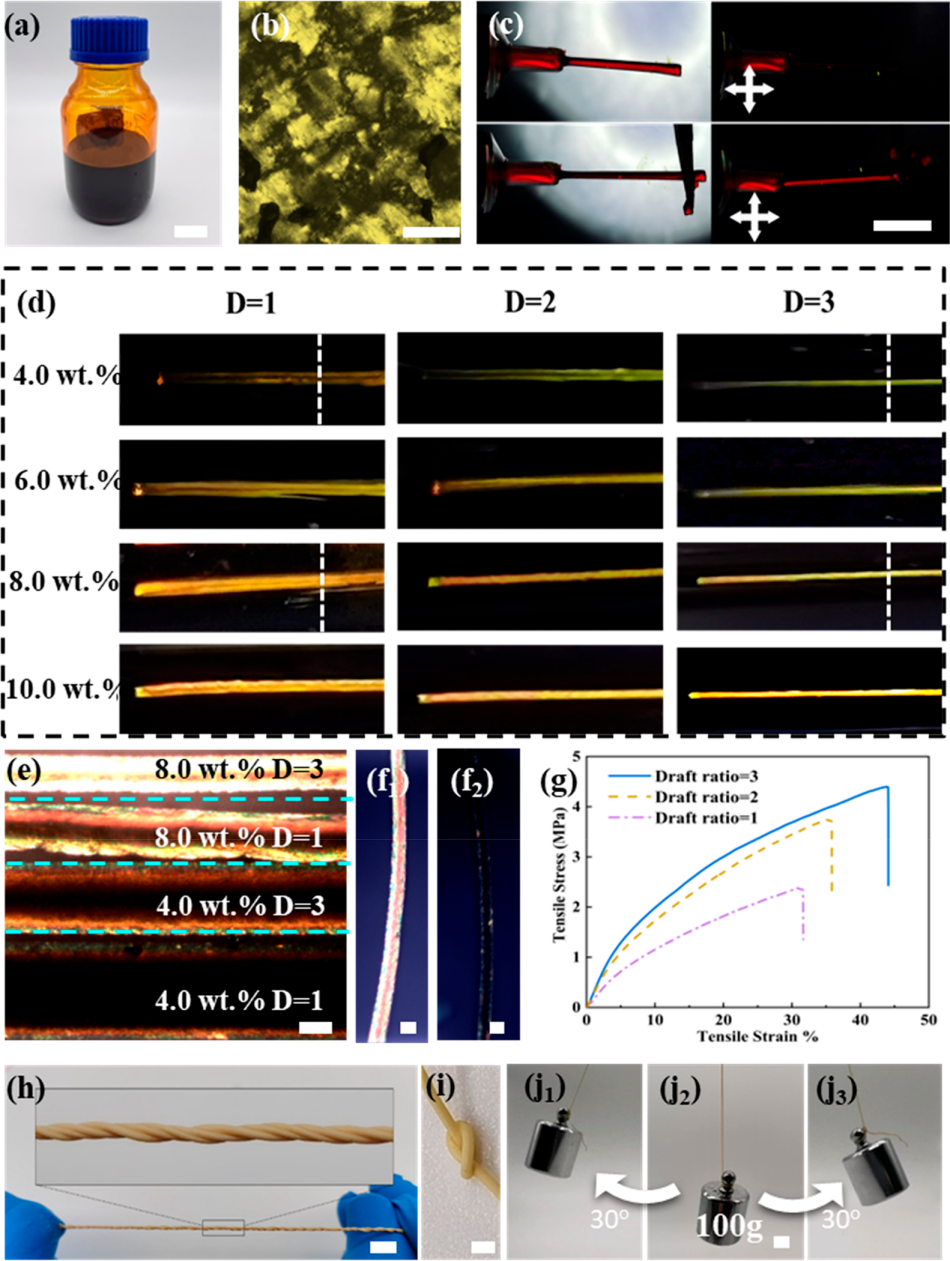
Fabricating process and performance test of NKLC gel fibers. (a) Photograph of the Kevlar nanofiber dispersion. Scale bar: 1 cm. (b) POM photograph of the Kevlar nanofiber dough at 8.0 wt %. Scale bar: 100 μm. (c) Digital photograph and polarized optical photograph of NKLC before (up) and after (down) drawing. Scale bar: 1 cm. (d) *In situ* POM photographs of Kevlar nanofiber with different concentrations and draft ratios during the liquid crystal spinning process. The white dotted lines represent the locations where relative brightness was measured with the ImageJ software. (e) POM photographs of the gel fibers with different concentrations and draft ratios. Scale bar: 100 μm. (f) POM photographs of the gel fiber with the concentration of 8.0 wt % and the draft ratio of 3.0 at 0° and 45° polarization. Scale bar: 100 μm. (g) Tensile stress–strain curves of the NKLC gel fiber with different draft ratios. (h) Photographs of the gel fiber undergoing twisting. Scale bar: 1 cm. (i) Photograph of the gel fiber undergoing knotting. Scale bar: 1 mm. (j) Photographs of the gel fiber hanging at a weight (100 g) and swinging at an amplitude of 60°. Scale bar: 1 cm.

The fabrication of the NKLC aerogel fiber starts from the preparation of Kevlar nanofiber dispersion (Figure 1a). The concentration of Kevlar nanofiber dispersion prepared with the methanol, potassium *tert*-butoxide, and DMSO system could reach 10.0 wt %, five times higher than that prepared with the potassium hydroxide-DMSO system.^31,32^ It is known that the p*K*_a1_ and p*K*_a2_ of Kevlar are approximately 19 and 29, respectively.^33^ According to the Brønsted-Lowry theory (or proton theory of acid–base),^34^ any base whose conjugate acid has a p*K*_a_ in DMSO greater than 29 would fully deprotonate Kevlar. Hence, the potassium *tert*-butoxide (*tert*-butyl alcohol as the conjugate acid, p*K*_a_ = 32) with DMSO can abstract protons from Kevlar to generate KNFs. Besides, the solubility of potassium *tert*-butoxide in DMSO is much higher than that of KOH in DMSO, and the addition of methanol further enhances the dissolution rate of Kevlar and reduces the viscosity of the dispersion, resulting in a higher concentration of Kevlar nanofibers.^33^

The rheological properties of Kevlar nanofiber dispersion with different concentrations (2.0, 4.0, 6.0, 8.0, and 10.0 wt %) were investigated, where the viscosities increase with the increase of the concentration of Kevlar nanofiber dispersion and reduce sharply under a high shear rate, especially for those with 8.0 and 10.0 wt % concentrations, relating to the formation of anisotropic domains in the dispersion (Figure S1). It should be noted that Kevlar nanofiber dispersion is a fluid state at a low concentration (2.0, 4.0, and 6.0 wt %), while becoming a dough state at a high concentration (8.0 and 10.0 wt %) at room temperature without shearing. According to Flory’s theory,^35^ the calculated critical concentration of the NKLC formation (C* = 8/*x*(1 – 2/*x*), where C* is the critical concentratio, and *x* is the aspect ratio, which is about 118, see Figure S2) is around 6.8 wt %, while that of the traditional Kevlar liquid crystal is 17.0 wt %.^36^ Above the critical concentration, the orientation of Kevlar nanofibers is no longer random. To pack more nanofibers in the dispersion, they are forced to align parallel to each other in the randomly oriented liquid crystalline domain. Since 6.8 wt % is the critical concentration of the NKLC formation, the 8.0 wt % Kevlar nanofiber dough should be in a liquid crystal state. To prove that, the polarized optical microscope (POM) photo of the 6.0–10.0 wt % Kevlar nanofiber dispersion was taken, as shown in Figure S3 (6.0, 7.0, 8.0, and 9.0 wt %) and Figure 1b (10.0 wt %). Under POM, at a low concentration (6.0 wt %), the visual field is completely black, indicating that the Kevlar nanofibers dispersion is in a disordered state at this time. As the concentration increases (7.0 wt %), bright spots appear in the field of view, and these bright spots can be regarded as the nucleation points of the alignment structure. This is similar to the birefringent spindles observed in nematic liquid crystals, formed by the agglomeration of ordered microdomains in the undesired state because the anchoring energy in the liquid crystal is greater than the surface tension. As the concentration further increased to 8.0 wt %, the nucleation points merged into bright regions, and a distinct schlieren texture appeared in the system. This texture is the characteristic optical texture of nematic liquid crystals, which also means that the liquid crystals formed are nematic liquid crystals. With a further increase in the concentration (9.0 wt %), a schlieren texture appeared in the entire region, and it can be concluded that a stable nematic liquid crystal was formed. If the concentration further increased, the texture structure would not change significantly, since the anisotropic domains can not expansion to long-range ordered structure. Therefore, the 8.0 wt % Kevlar nanofiber dough was selected as the representative NKLC, except for a special claim. The Kevlar nanofiber dispersions of different concentrations were quickly frozen in liquid nitrogen to protect the original dispersion state and arrangement state, and then, the solvent was removed by freeze-drying to obtain Kevlar nanofiber cryogel blocks, which can show the arrangement state of nanofibers in the Kevlar nanofiber dispersion solution. In the cross sections of cryogel prepared from 6.0 wt % Kevlar nanofiber dispersions (Figure S4a,b), it can be found that the arrangement of Kevlar nanofibers is disordered, while for that from 10.0 wt % Kevlar nanofiber dispersions (Figure S4c,d), the Kevlar nanofibers start to form domains with different orientations.

It is normal for liquid crystals to be oriented under the action of external force.^12,37^ To determine the building block orientation during transportation and extrusion, POM was utilized to observe the NKLC flowing in a transparent capillary. There is no obvious building block orientation when transporting at low speed (<1.0 cm·s^–1^) in a thick pipe with a diameter of 5.0 mm, while an obvious building block orientation occurs when transporting at high speed (15.0 cm·s^–1^) in a thin tube with a diameter of 1.0 mm (Figure S5). Besides, the polarized lens group is applied to investigate the building block orientation of the NKLC before and after drawing. As shown in Figure 1c, the undrafted NKLC dough appears dark red under normal light and relatively dim under polarized light, which is achieved by the angles of the polarizer and analyzer being shifted from 0 to 90°, while the brightness is significantly enhanced under polarized light after drawing. This phenomenon is due to the directional arrangement of the Kevlar nanofiber under the drafting effect, forming a long-range ordered structure that allows polarized light to pass through.^38,39^

In the liquid crystal spinning process, the NKLC was extruded into a coagulation bath (*i*.*e*., water). The gel fibers were formed from the NKLC by capturing protons from water to form hydrogen bonds, which are illustrated in Figure S6. The building block orientation of gel fibers prepared with different concentrations of Kevlar nanofiber dispersions and different draft ratios during the liquid crystal spinning were monitored with an *in situ* orientation detection setup (Figure S7). As shown in Figure 1d, the brightness of the gel fibers under polarized light dramatically increases with increasing the Kevlar nanofiber dispersion concentration and/or the spinning draft ratio, and the brightness reaches the maximum at the highest concentration of 10.0 wt % with the highest draft ratio of 3.0. Actually, from empirical investigations, the maximum draft ratio during the liquid crystal spinning of 10.0 wt % NKLC is about 3.6. However, when the draft ratio approaches the maximum one, the gel fibers are fragile. So, a draft ratio of 3.0 is preferred for liquid crystal spinning of NKLC. To quantitatively determine the brightness, ImageJ software was applied to analyze the polarized optical photos of gel fibers prepared with different concentrations and different draft ratios (Figure 1e). This is due to the fact that, the higher the degree of orientation of the fiber, the higher the intensity of light polarized at that location, resulting in an increase in the brightness of the fiber. As shown in Figure S8, the relative brightness increased from 0.28 for the gel fiber prepared with 4.0 wt % Kevlar nanofiber dispersion and the draft ratio of 1.0–0.82 for that prepared with 8.0 wt % Kevlar nanofiber dough and a draft ratio of 3.0. Figure 1f shows the photos of the gel fiber obtained from 8.0 wt % Kevlar nanofiber dough with a draft ratio of 3.0 under the polarized light incidence at 0° and 45°, respectively, which exhibit the obvious contrast between bright and dark, indicating the axially oriented structure.

In addition to the special optical properties, the axially oriented structure will bring excellent mechanical strength. The tensile properties of the liquid crystal gel fiber prepared from 8.0 wt % Kevlar nanofiber dough are shown in Figure 1g, where the tensile strength and elongation at break increase significantly from 2.2 to 4.2 MPa and 32% to 45% as the draft ratio increases from 1.0 to 3.0. Meanwhile, the mechanical strength of the gel fibers improves with the increase of the concentration and reaches 5.0 MPa when the Kevlar nanofiber concentration is 10.0 wt % at a draft ratio of 3.0 (Figure S9), which is superior or comparable to that of most previous reported gel fibers,^40−43^ including bacterial cellulose-gelatin double-network hydrogel fibers (with a mechanical strength of 3.0 MPa) and poly(2-acrylamido-2-methylpropanesulfonic acid)/polyacrylamide hydrogel fibers (with a mechanical strength of 5.6 MPa). The gel fiber prepared with 8.0 wt % Kevlar nanofiber dough is strong enough to be densely twisted (Figure 1h) and exhibits high flexibility (with a curvature radius of ca. 500 μm) to tie the knot (Figure 1i). Moreover, it can hang 100 g weight and swing (Figure 1j) or rotate (Movie S1) at an amplitude of 60°.

### Performance Test of the NKLC Aerogel Fibers

**Figure 2:**
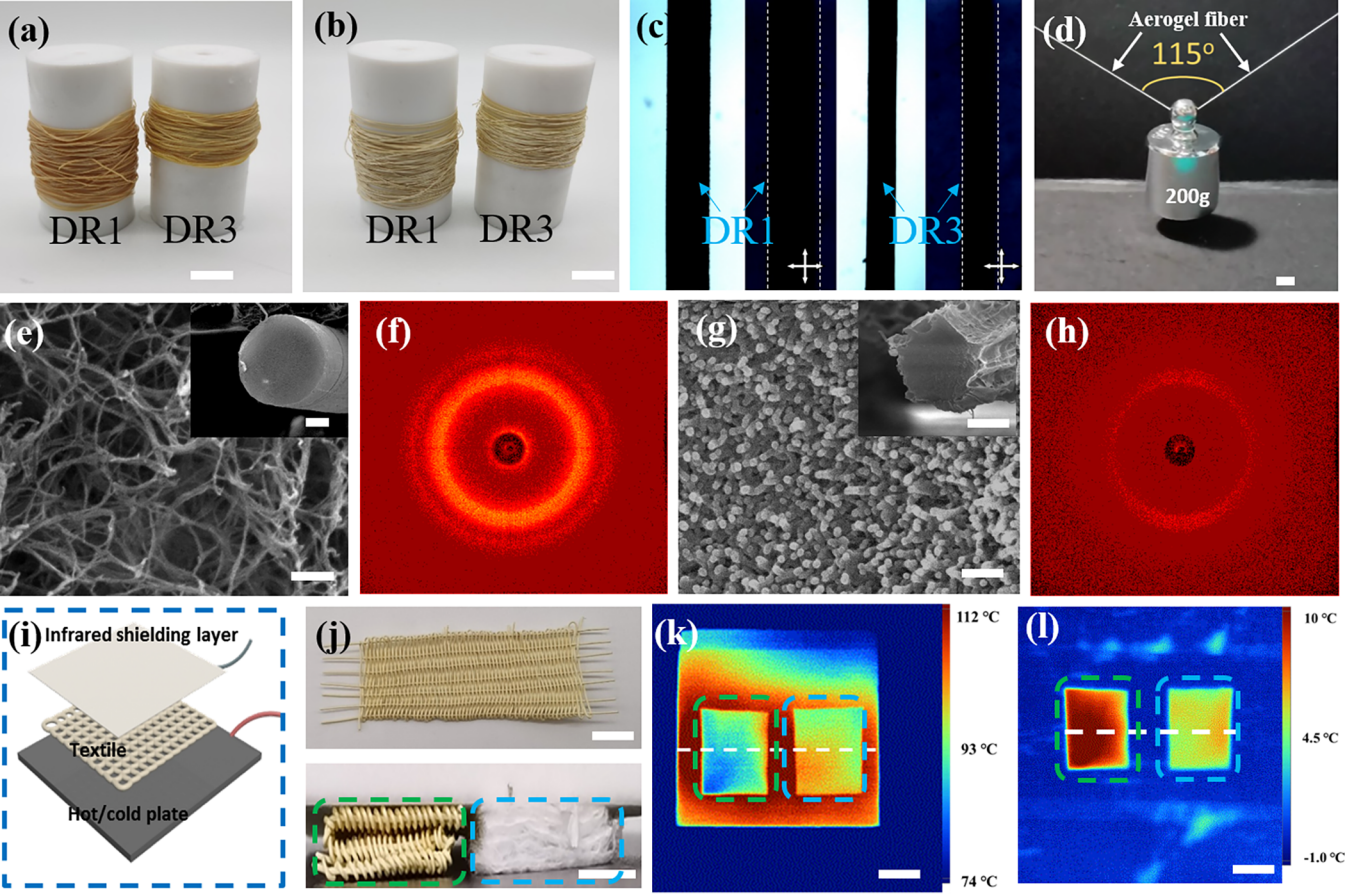
Performance test of the NKLC aerogel fibers. (a) Photograph of DR1 gel fiber (left) and DR3 gel fiber (right). Scale bar: 1 cm. (b) Photograph of DR1 aerogel fiber (left) and DR3 aerogel fiber (right). Scale bar: 1 cm. (c) Optical microscopy photographs of DR1 aerogel fiber (left) and DR3 aerogel fiber (right) under normal light and polarized light, respectively. (d) Digital photograph of DR3 aerogel fiber hanging a weight (200 g). Scale bar: 1 cm. (e) Cross-section SEM image of DR1 aerogel fiber with a scale bar of 500 nm. The inset is its SEM image under low magnification with a scale bar of 10 μm. (f) WAXS pattern of the DR1 aerogel fiber. (g) Cross-section SEM image of DR3 aerogel fiber with a scale bar of 500 nm. The inset is its SEM image under low magnification with a scale bar of 10 μm. (h) WAXS pattern of DR3 aerogel fiber. (i) Schematic diagram of the experimental setup for thermal insulation measurement. (j) Digital photograph of the DR3 aerogel fiber textile (upper) for thermal insulation test. Scale bar: 1 cm. Digital photographs of the DR3 aerogel fiber mat and the hollow cotton fiber mat (lower). Scale bar: 1 cm. (k) Thermal infrared image of the DR3 aerogel fiber mat (left) and the hollow cotton fiber mat (right) under 100 °C at an equilibrium state. Scale bar: 1 cm. (l) Thermal infrared image of the DR3 aerogel fiber mat (left) and the hollow cotton fiber mat (right) under 0 °C at an equilibrium state. Scale bar: 1 cm.

Although the brightness of the NKLC gel fibers prepared with a draft ratio (DR) of 1.0 (named DR1) and a draft ratio of 3.0 (named DR3) are quite different under polarized light, they present negligible differences in appearance under normal light (Figure 2a). For the corresponding liquid crystal aerogel fibers, it becomes more difficult to distinguish the difference under normal light (Figure 2b). The liquid crystal aerogel fibers are opaque to both normal light and polarized light (Figure 2c). Notably, in order to exactly evaluate the optical properties, DR1 and DR3 are almost identical in diameter (120 μm), which was obtained by regulating the diameter of spinning needles. Because the refractive index contrast (Δ*n* = |*n*_KNF_ – *n*_air_|, where Δ*n* is the refractive index contrast, *n*_KNF_ is the refractive index of KNF, which is 1.3, and *n*_air_ is the refractive index of air, which is 1.0) across the Kevlar nanofiber-pore boundaries is large, it causes the pores to efficiently scatter both natural and polarized light. When the NKLC aerogel fibers change into gel fibers, the transparency will increase accordingly, due to the refractive index contrast within the gel fiber (Δ*n* = |*n*_KNF_ – *n*_water_ | = |1.3 – 1.33| = 0.03, or Δ*n* = |*n*_KNF_ – *n*_EtOH_ | = |1.3 – 1.36| = 0.06, where *n*_water_ and *n*_EtOH_ are the refractive index of water and ethanol, respectively) being 1 order of magnitude smaller than that of within the aerogel fiber. This phenomenon has been reported elsewhere.^44^

The mechanical properties of aerogel fibers fabricated with different concentrations of Kevlar nanofiber and different draft ratios were measured, and the results are shown in Figure S10. It is worth mentioning that both tensile strength and elongation at break improved significantly with the increase of the draft ratio. Because as the draft ratio increases, the number of nanofibers aligned along with the axial direction increases, resulting in simultaneous increases in the breaking strength and elongation at the break (Figure S11).The maximum tensile strength of the NKLC aerogel fibers can reach 41.0 MPa, which is the highest among various aerogel fibers (Figure S10c), including polyimide aerogel fibers (with a maximum tensile strength of 11.0 MPa)^11^ and graphene aerogel fibers (with a maximum tensile strength of 1.45 MPa).^13^ Attributing to the high mechanical strength, a single NKLC aerogel fiber can withstand a tensile load of 200 g at a flat angle of 115°, as shown in Figure 2d. Surprisingly, the specific surface area of the NKLC aerogel fiber remains high, fluctuating between 204 and 245 m^2^/g (Figure S12), which is not available in Kevlar fibers (Figure S13a–c). Besides, the specific surface area has no correlation with orientation degree (Figure S12). It is well-known that supercritical CO_2_ (ScCO_2_) drying is the classic way to fabricate aerogels from gels. For comparison, the aerogel fibers prepared *via* the traditional ScCO_2_ drying are conducted for mechanical property measurement and microscopic pore structure characterization as well. The results are shown in Figure S14, where the tensile strain of the NKLC aerogel fibers prepared *via* the freeze-drying is slightly higher than that fabricated *via* the ScCO_2_ drying, and the specific surface area of the NKLC aerogel fibers prepared *via* the freeze-drying is a little lower than that prepared *via* the ScCO_2_ drying, attributing to slight shrinkage in the radial direction. But overall, both freeze-drying and ScCO_2_ drying can maintain the porous structure perfectly. The freeze-drying was chosen preferentially mainly due to the convenient operation.

To explore the relationship between properties and structures, scanning electron microscopy (SEM) and wide-angle X-ray scattering (WAXS) are applied to get insight into the microstructures of the NKLC aerogel fiber. The cross-section of DR1 aerogel fiber is almost perfectly round, and the micromorphology shows an interconnected three-dimensional nanofibrillar network (Figure 2e). From the cross-section of the undrawn fiber (Figure S15a), domains with different orientations can be observed, which are inherited from the microdomain structure of the liquid crystal structure of the Kevlar nanofiber dispersion. Correspondingly, the interface diffraction aperture of the DR1 aerogel fiber presents a uniform ring shape (Figure 2f), and the calculated orientation degree is only 0.08, while the SEM image of the DR3 aerogel fiber shows that the Kevlar nanofibers are arranged neatly in the direction perpendicular to the cross-section (Figure 2g), which means that the nanofibers are oriented along the axial direction. Besides, the aerogel fiber shrinks in the radial direction, resulting in an irregular surface (Inset in Figure 2g). On the cross-section of the DR3 fiber (Figure S15b), the microdomain structure of the liquid crystal dispersion structure was not observed, and the nanofibers were neatly arranged in the vertical direction of the cross-section (Figure 2h). Therefore, the number of nanofibers that provide structural support in the radial direction decreases after the orientation, thus causing shrinkage in the radial direction during the drying process (inset in Figure 2h). Moreover, the shrinkage ratio of the aerogel fibers increases with the rise of the draft ratio, which also illustrates the orientation effect of the draft on the nanofibers (Figure S16). Correspondingly, the interface diffraction aperture of the DR3 aerogel fiber is oriented, and the degree of orientation is 0.36. In order to visually compare the difference in fiber orientation, the WAXS scanning intensity-azimuthal angle curve of the fibers is normalized and compared in Figure S17, and it can be found that the orientation degree of DR3 fiber is significantly higher than that of the DR1 fiber.^45,46^ Furthermore, small-angle X-ray scattering (SAXS) was also applied to inspect the orientation of the building blocks. As shown in Figure S18, the diffraction ring of DR1 fibers is circular, while that of DR3 fibers is elliptical, which indicates that the higher draft ratio results in a higher orientation, confirming that draft ratios contribute to the building block orientation. The thermal weight loss curve of Kevlar fiber has been added for comparing with that of Kevlar aerogel in Figure S13d, which shows that there is no chemical modification occurs during the aerogel preparation process

Thermal insulation, as the most important property of the aerogel fibers, has been comprehensively investigated. The schematic diagram of the measurement device is shown in Figure 2i, where the textile is placed on a cold/hot plate, and covered with a masking tape as an infrared shielding layer to eliminate the effect of emissivity in the radiation temperature measurement. Benefiting from the high mechanical strength, the DR3 aerogel fibers can be woven into textile and laminated into a mat with an areal density of 46 g/m^2^. The hollow cotton fiber mat as the representatively commercial thermal insulation material with an areal density of 150 g/m^2^ at the same thickness was selected for comparison with our aerogel fiber mat (Figure 2j). To evaluate the thermal insulation under high temperatures, these two mats were placed on a ceramic electric heating plate with a temperature of 100 °C, and the infrared image is shown in Figure 2k. The lowest surface temperature of the DR3 aerogel fiber mat is 82 °C, and the average is 90 °C, while the lowest surface temperature of the hollow cotton fiber mat is 90 °C and the average is 95 °C (Figure S19). To determine the thermal insulation performance under low temperature, these two mats were placed on a cold plate (0 °C) with an internally circulated low-temperature liquid. In order to avoid the condensation of water vapor, the surface was covered with a hydrophobic layer. Similar to the measurement under high temperature, the DR3 aerogel fiber mat demonstrated better thermal insulation performance than that of the cotton hollow fiber mat (Figure 2l). The average surface temperature of the DR3 aerogel fiber mat is 9.5 °C, and that of the cotton hollow fiber mat is 6 °C (Figure S20). These results are consistent with the thermal conductivity of the DR3 aerogel fiber mat and the cotton hollow fiber mat, which are 0.037 and 0.041 W·m^–1^·K^–1^, respectively. Therefore, the textile woven with the DR3 aerogel fiber has excellent thermal insulation performance at a wide temperature range. Besides, the thermal insulation performance of the textile woven with DR1 aerogel fiber was compared with that of DR3 aerogel fiber, and the infrared image is shown in Figure S21, which displays similar thermal insulation performance. This illustrates that the building block orientation structure of the fiber has no significant effect on the radial heat insulation performance. The thermal insulation property of the NKLC aerogel fiber fabricated with ScCO_2_ was compared with that of the DR3 aerogel fiber (fabricated with freeze-drying). The infrared image also exhibits that they have a similar thermal insulation performance, attributing to the almost identical microstructure (Figure S22). However, the thermal insulation performance of the NKLC aerogel fibers is sensitive to moisture. It would decrease significantly in a high humidity environment due to the intrinsic hydrophilicity of the Kevlar and the porous structure, providing a large number of capillary channels to capture moisture. Besides, the mechanical properties of the NKLC aerogel fibers also decline sharply with humidity increases (Figure S23).

### Superhydrophobic Functionalization of the NKLC Aerogel Fibers

**Figure 3:**
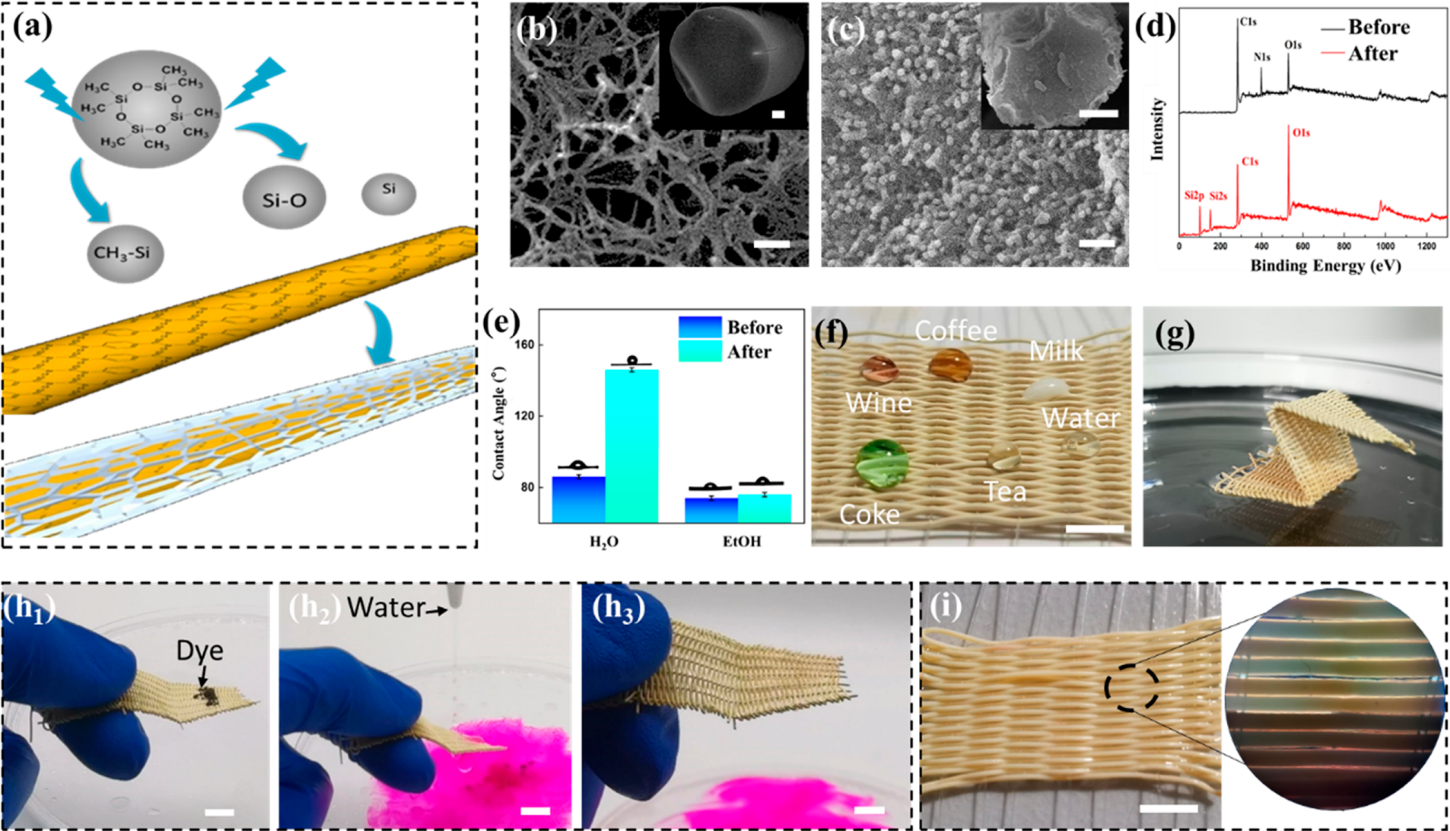
Superhydrophobic functionalization of the NKLC aerogel fibers. (a) Schematic diagram of the hydrophobic functionalization of NKLC aerogel fiber *via* cold plasma treatment. (b) Cross-section SEM image of the hydrophobic DR1 aerogel fiber with a scale bar of 500 nm. The inset is its low magnification image with a scale bar of 100 μm. (c) Cross-section SEM image of the hydrophobic DR3 aerogel fiber with a scale bar of 500 nm. The inset is its low magnification image with a scale bar of 100 μm. (d) XPS pattern of the NKLC aerogel fiber before and after hydrophobic functionalization. (e) Water and ethanol contact angles of the NKLC aerogel fibers textile before and after hydrophobic functionalization. (f) Photograph of the state of various types of liquid droplets on the hydrophobic textile of NKLC aerogel fibers. Scale bar: 1 cm. (g) Photograph of the hydrophobic textile of NKLC aerogel fibers that float on the water. (h) Photographs show that the hydrophilic dye can be washed off easily from the hydrophobic textile of NKLC aerogel fibers with water without any liquid residue. Scale bar: 1 cm. (i) Photograph (left) and POM photo (right) of DR1 and DR3 hybrid textile after infiltration with ethanol. Scale bar: 1 cm.

In order to prevent the degradation of mechanical and thermal insulating performances under a humidity environment, the cold plasma technology was creatively applied to the hydrophobic functionalization of the NKLC aerogel fibers, which is schematically illustrated in Figure 3a. Octamethylcyclotetrasiloxane (D4) was selected from various hydrophobic agents, attributing to that it could endow the optimal hydrophobicity (Table S1). The cross-sectional SEM images of DR1 and DR3 aerogel fiber after cold plasma treatment are shown in Figure 3b,c, respectively, which reveal that the hydrophobic functionalization only has an insignificant impact on the porous structure (Figure S24). However, a large number of particles with an average diameter of 2 nm appeared on the surface of the Kevlar nanofibers after hydrophobic functionalization, indicating that the small molecules obtained by the decomposition of D4 can enter the aerogel fiber and polymerize on the surface of the Kevlar nanofibers. It is worth mentioning that the hydrophobic functionalization *via* cold plasma treatment has a negligible impact on the mechanical strength and thermal insulation performance of the NKLC aerogel fibers (Figures S25 and S26). From the TG curves (Figure S27), it can be observed that the cold plasma treated fibers have obvious mass loss compared to the untreated fibers at around 500 °C, and the final residual mass is lower than that of the untreated fibers. These all indicate that the cold plasma treatment makes the hydrophobic polymer adhere to the fiber surface. Furthermore, the surface components of the aerogel fibers were characterized by X-ray photoelectron spectroscopy (XPS). The presence of Si in the XPS spectra after hydrophobic functionalization confirms the hydrophobic layer on the aerogel fiber surface (Figure 3d). The water and ethanol repelling properties were investigated by measuring the water contact angle and ethanol contact angle, respectively, and the results are shown in Figure 3e. After the hydrophobic functionalization, the contact angle to water increased from 82° to 154°, which means that the aerogel fiber changed from hydrophilic to superhydrophobic. Meanwhile, the water contact angles of fibers with different building block orientation degrees before and after hydrophobic functionalization are compared, and it was found that the orientation degree has a negligible effect on the hydrophobicity (Table S2). However, the contact angle to ethanol does not increase observably after cold plasma treatment (Figure 3e). Therefore, it can be concluded that the surface tension of the NKLC aerogel fibers (KLCAF) is smaller than that of water but higher than that of ethanol (*i*.*e*., σ_water_ > σ_KLCAF_ > σ_ethanol_, where σ_water_ is the surface tension of water, σ_KLCAF_ is the surface tension of KLCAF, amd σ_ethanol_ is the surface tension of ethanol). The textile woven from the hydrophobic NKLC aerogel fibers can prevent the immersion of most liquids in daily life, such as water, tea, milk, red wine, coffee, and coke (Figure 3f). Besides, this hydrophobic aerogel fiber textile can float on the water surface without getting wet (Figure 3g), and the hydrophilic dye (*e*.*g*., rhodamine B) on the surface can be washed off easily with water without any liquid residue, showing the excellent self-cleaning performance (Figure 3h and Movie S2). On account of the contact angle of the fiber to ethanol being less than 90°, the NKLC aerogel fiber textile can absorb ethanol or ethanol aqueous solution to convert into an alcogel fiber textile. When it is taken out, the ethanol will volatilize from the alcogel fiber textile without triggering the collapse of the aerogel structure, due to the low surface tension (Table S1). Therefore, this NKLC aerogel fiber textile can transfer between aerogel–gel cyclically with ethanol absorbing and ambient pressure drying (Figure S28). Furthermore, a textile woven with DR1 and DR3 can be identified after infiltration with ethanol (Δ*n* = |*n*_KNF_ – *n*_EtOH_ | = |1.3 – 1.36| = 0.06, where *n*_EtOH_ is the refractive index of ethanol) under polarized light, because of the different brightness (Figure 3i).

### Information Storage and Decryption Function of Digital Textile

**Figure 4:**
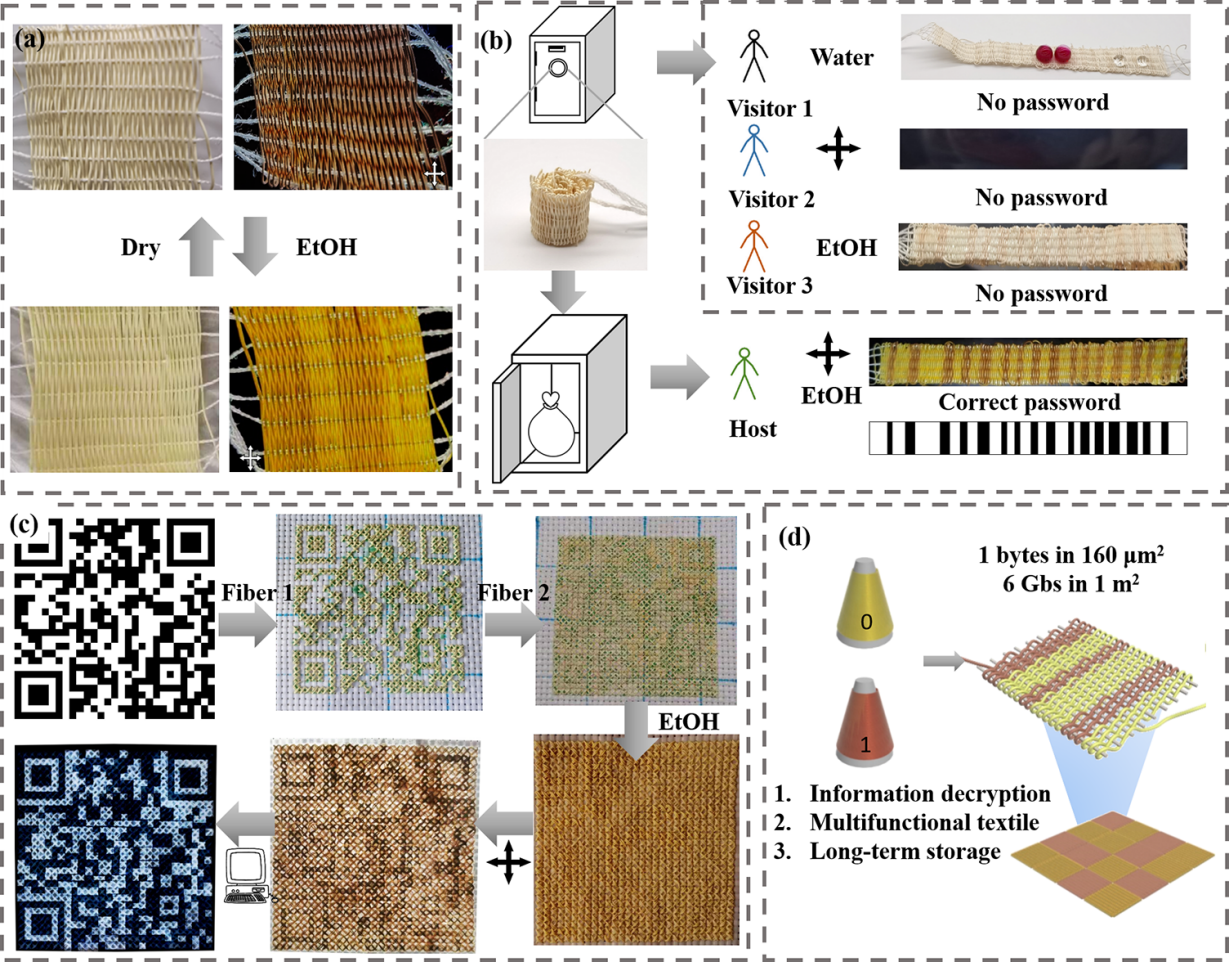
Information storage and decryption function of the digital textile. (a) Photographs and polarizing photographs of the NKLC aerogel fiber textile transferred between aerogel-gel cyclically with ethanol absorbing and ambient pressure drying, where the gel textile displays light and dark stripes under polarized light. (b) Schematic diagrams and photographs to illustrate the process of information encryption and on-demand decryption of a NKLC aerogel fiber based barcode. (c) Photographs and polarizing photographs to illustrate a two-dimensional code mode was encrypted and on-demand decrypted with the NKLC aerogel fiber embroidery. (d) Imaging diagram of the information storage textile.

Given the aforementioned exploration, a digital textile was obtained by rationally weaving DR1 and DR3 aerogel fibers. The diameter of these fibers is 120 μm, which is lower than the critical one (121.4 μm, calculated according to the previous report) for wearing comfort.^20^ The resulting hybrid aerogel textile is uniform under normal light and polarized light. When impregnating in ethanol, it would transform from an aerogel textile into a gel textile. As expected, the gel textile is still uniform under normal light but exhibits bright and dark stripes under polarized light. More importantly, the superhydrophobic hybrid aerogel textile can be transferred between aerogel–gel at least 10 cycles with ethanol absorbing and ambient pressure drying in sequence (Figure S28), showing the great potential application in information encryption and on-demand decryption (Figure 4a). The specific surface area of the aerogel fiber after 10 aerogel-gel cycles is 177 m^2^/g, which is only slightly lower than that of the original one with 204 m^2^/g (Figure S29a). Besides, the cross-sectional SEM images show that the outer layer of the fiber after 10 aerogel-gel cycles shrinks, but the interior retains the porous network structure (Figure S29b,c). Therefore, an identification barcode (*i*.*e*., information) was designed and fabricated with DR1 and DR3 aerogel fibers to visually display the information encryption, storage, and decryption. As shown in Figure 4b, the digital textile does not display the stored information under both normal light and polarized light. When impregnating the digital textile with ethanol, the information is still hidden under normal light. It only can be identified when the digital textile is both in a gel state and under polarized light, which also can be read by a computer (Movie S3). Furthermore, a digital textile with a two-dimensional code (serving as a hidden password) has been fabricated by embroidering with DR1 and DR3 aerogel fibers. Similarly, the two-dimensional code only can be shown through the decryption processes, which are (1) immersing in ethanol and (2) observing under polarized light. Then, the accurate two-dimensional code can be obtained by image processing (Figure 4c).

Therefore, an information storage textile scheme has been proposed, where two kinds of aerogel fibers with different building block orientation degrees are used to represent 0 and 1, and every eight aerogel fibers constitute a byte (Figure 4d). The length of one byte is the sum of the diameter of eight fibers, and the width was calculated according to the weaving style. If the fiber diameter is 10 μm and the width of a byte is 2 μm then each square centimeter of the information textile can store 625,000 bytes, which means that up to 6.0 Gb of information can be stored in one square meter. Therefore, a large amount of information can be stored in the digital textile. In addition, the digital textile inherits the intrinsic stability under high/low temperatures, which can withstand high temperature (200 °C) or low temperature (−196 °C, liquid nitrogen) treatment, without degrading the performance of information encryption and on-demand decryption (Figure S30). Compared with other information encryption carriers, such as photochromic materials,^47^ fluorescent materials,^48^ and luminescent materials,^49^ the NKLC aerogel fibers exhibit significant advantages including (1) the binary format of information encryption and (2) the high security during transmission, attributing to the stability under high/low temperature and the two-step for decryption. Therefore, the NKLC aerogel fibers can provide effective information encryption, secure storage, and on-demand decryption, which can play a vital role in the information era.

## Conclusions

In summary, the NKLC gel/aerogel fibers with controllable building block orientation have been realized *via* corresponding liquid crystal spinning with the proton-donator solvent as the coagulation bath. The NKLC gel fibers with different building block orientation display distinguishable brightness (from 0.28 to 0.82 in relative brightness) under polarized light, which have been fabricated by adjusting the concentration of Kevlar nanofiber and draft ratio during the dynamic sol–gel transition process. The corresponding NKLC aerogel fibers exhibit extremely high mechanical strength (41 MPa), outstanding thermal insulation performance (0.037 W·m^–1^·K^–1^), as well as superhydrophobic properties (with a water contact angle of 154°). Besides, the textile woven from these NKLC aerogel fibers can be transferred between aerogel-gel cyclically by absorbing ethanol and ambient pressure drying alternately. Therefore, digital textiles with encrypted information (*i*.*e*., either bar codes or two-dimensional codes) have been realized by weaving or embroidering with these NKLC aerogel fibers with different building block orientation degrees. The encrypted information only can be decrypted when immersing the digital textile in ethanol and observing it under polarized light, showing excellent information security. Thus, these NKLC aerogel fibers demonstrate great potential for applications in thermal insulation, information encryption, on-demand decryption, *etc*.

## Methods

### Materials

Kevlar-29 1000D was purchased from DuPont Company in Wilmington, Delaware. Dimethyl sulfoxide (DMSO, 99%) and anhydrous ethanol (99.5%) were obtained from China National Pharmaceutical Group Co., Ltd. (Sinopharm). Anhydrous methanol (99%), potassium *tert*-butoxide (98%), and *tert*-butanol (99%) were purchased from Aladdin Company. Deionized water (with a resistivity of 18.2 MΩ·cm^–1^) was obtained from a Milli-Q system (Millipore).

### Fabrication of the Nanoscale Kevlar Liquid Crystal (NKLC)

The Kevlar nanofiber dispersions with different concentrations (2.0, 4.0, 6.0, 8.0, and 10.0 wt %) were prepared *via* a “one-pot” method. Specifically, 10.0 g of Kevlar 1000D and 10.0 g o fpotassium *tert*-butoxide were added to the refined DMSO with rapidly stirring for 10 min. Then, 10.0 g of anhydrous methanol was added in three batches within 1 h, which were kept stirring for another 3–8 h until the sample is homogeneous. The obtained Kevlar nanofiber dispersion exhibited liquid-crystalline behavior at 8.0 and 10.0 wt %.

### Fabrication of the NKLC Aerogel Fibers

The Kevlar nanofiber dispersion with different concentrations was extruded from a pump-controlled syringe into a coagulation bath (*i*.*e*., water) with different collection linear velocities. The ratio of the collection linear velocity to the extrusion linear velocity is defined as the draft ratio, and the draft ratio was controlled under 3.0. After gelation, the fibers were collected with a polytetrafluoroethylene (PTFE) collecting device, and the NKLC hydrogel fibers were obtained after washing away the residual coagulation solution. Then, the obtained hydrogel fibers were immersed in a 25% *tert*-butyl alcohol aqueous solution for solvent replacement. Subsequently, the gel fibers were frozen under −20 °C and freeze-dried for more than 24 h under 0.05 mbar. Finally, the NKLC aerogel fibers with different building block orientations were obtained. For further application of these aerogel fibers, a hand knitting machine was used to weave the fibers into textiles.

### Cold Plasma Treatment

Cold plasma treatment was performed with an HD-1A/B cold plasma modification processor (Changzhou Zhongke Changtai Plasma Technology Co., Ltd.). The sample was put into the chamber, which was then evacuated to 5 Pa. Subsequently, argon was filled into the chamber until the vacuum degree reached 80 Pa, followed by discharging at 80 W for 90 s. Then, the chamber was evacuated to 5 Pa, fed D4 to 20 Pa, and discharged at 80 W for 90 s in sequence. The above process was repeated four times, and then, the sample was taken out.

### Characterization

The morphology of NKLC aerogel fibers was observed by scanning electron microscopy (SEM, Hitachi S-4800) at an acceleration voltage of 10–20 kV. To enhance the electrical conductivity, gold deposition was applied on the samples before SEM testing. The pore structure and pore size distribution of the NKLC aerogel fibers were analyzed with a nitrogen adsorption and desorption instrument (ASAP 2020, Micromeritics) with the Barrett–Joyner–Halenda (BJH) nitrogen adsorption and desorption method. The specific surface area of the aerogel fibers was determined by the Brunauer–Emmett–Teller (BET) method at 77 K, based on the amount of N_2_ adsorbed at pressures 0.05 < *P*/*P*_0_ < 0.3. Mechanical properties were tested by the tensile mode of an electronic universal testing machine (Instron 3365) with a gauge length of 10 mm at a loading rate of 1 mm/min. The thermogravimetric (TG) analysis was conducted with NETZSCH TG 209F1 Libra at a heating rate of 10 °C·min^–1^ in a nitrogen flow. The wide-angle X-ray scattering (WAXS) was performed on an X-ray scatterometer NanoSTAR (Bruker-AXS). The infrared thermal images were taken by an infrared camera (Fluke TiX580) and analyzed with Smart View. The heat source was an electric heating ceramic plate with a rated voltage of 12 V and a rated power of 30 W. The maximum temperature in the testing process was 120 °C, and the equilibrium one was 110 °C. The cryogenic source was provided by a cryogenic cycle machine (DLSB-ZC, Zhengzhou Great Wall Science, Industry and Trade Co., Ltd.), and anhydrous ethanol was adopted as the circulating cryogenic liquid.

## Supporting Information Available

Figures of viscosity-shear rate curve, TEM and SEM images, POM images, schematic illustration of the orientation of NKLC during the extrusion process, schematic diagram of the sol-gel transition process to obtain the NKLC gel fibers, schematic diagram and photograph of *in situ* orientation detection equipment during liquid crystal spinning process, mechanical tensile strength, tensile stress–strain curves, nitrogen adsorption–desorption isotherms, TG curves, WAXS scanning intensity-azimuthal angle curve, SAXS patterns, temperature distribution profiles, and infrared photos, and tables of contact angle to water of the aerogel fibers prepared with a different hydrophobic agent during cold plasma treatment and contact angle to water of the aerogel fibers prepared with different Kevlar nanofiber concentrations and different draft ratios (PDF)
Movie of NKLC gel fiber hanging 100 g weight and rotate at an amplitude of 60° (MP4)
Movie of self-cleaning of the aerogel fiber (MP4)
Movie of using a mobile phone to read the information on the decrypted digital fabric (MP4)
