# Supplementary material for: Nanoscale Kevlar Liquid Crystal Aerogel Fibers
Source: ACS Nano. 2022 Sep 2;16(9):15237–48. doi: 10.1021/acsnano.2c06591 (PMC9527790; doi:10.1021/acsnano.2c06591)
Supplement: Supplementary file 1 — nn2c06591_si_001.pdf [file nn2c06591_si_001.pdf]

Nanoscale Kevlar Liquid Crystal Aerogel Fibers

Zengwei Liu[a,b], Jing Lyu[b], Yi Ding[b], Yaqian Bao[a,b], Zhizhi Sheng[b], Nan Shi[b] and Xuetong Zhang[b,c]*

[a] School of Nano-Tech and Nano-Bionics, University of Science and Technology of China, Hefei, 230026, P. R. China.

[b] Suzhou Institute of Nano-tech and Nano-bionics, Chinese Academy of Sciences, Suzhou, 215123, P. R. China.

[c] Division of Surgery and Interventional Science, University College London, London, NW3 2PF, United Kingdom.

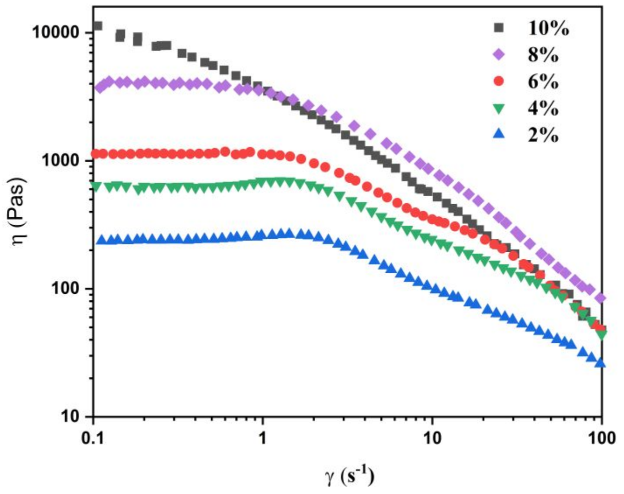

Figure S1. Viscosity as a function of shear rate of the Kevlar nanofiber dispersions with different concentrations.

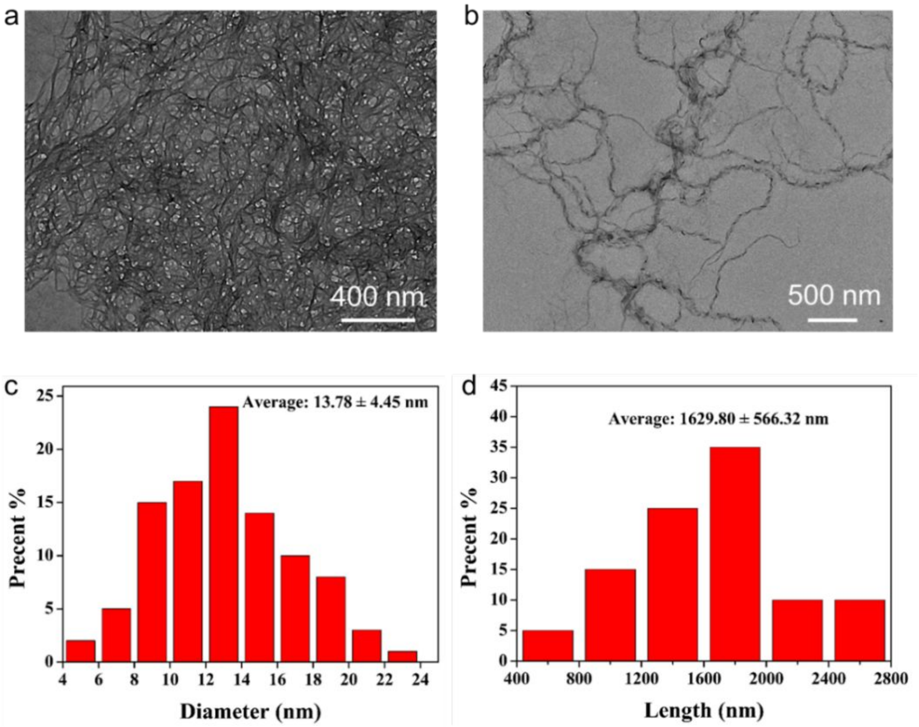

Figure S2. (a-b) Transmission electron microscope images of Kevlar nanofibers with different magnification. (c) The average diameter (d) distribution of Kevlar nanofibers. (d) The average length (l) distribution of Kevlar nanofibers. The aspect ratio (x=l/d) is ca. 118.

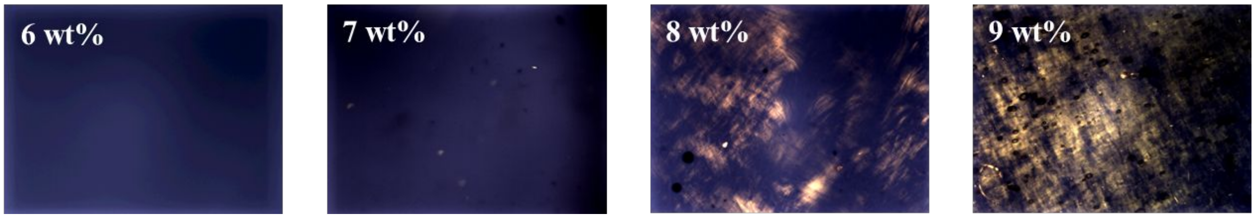

Figure S3. The polarized optical microscope (POM) photos of the Kevlar nanofiber dispersion with a concentration of 6.0 wt.% to 9.0 wt.%.

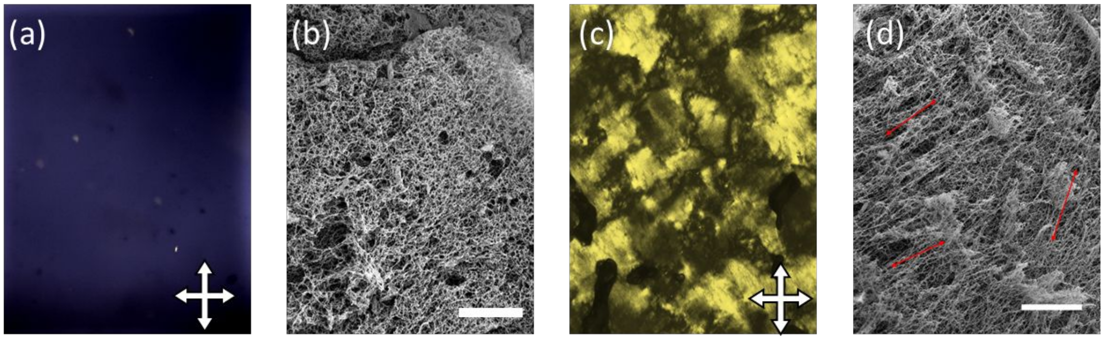

Figure S4. (a) The POM photo of the 6.0 wt.% Kevlar nanofiber dispersion, and (b) the SEM image of its corresponding cryogel. (c) The POM photo of the 10.0 wt.% Kevlar nanofiber dispersion, and (d) the SEM image of its corresponding cryogel.

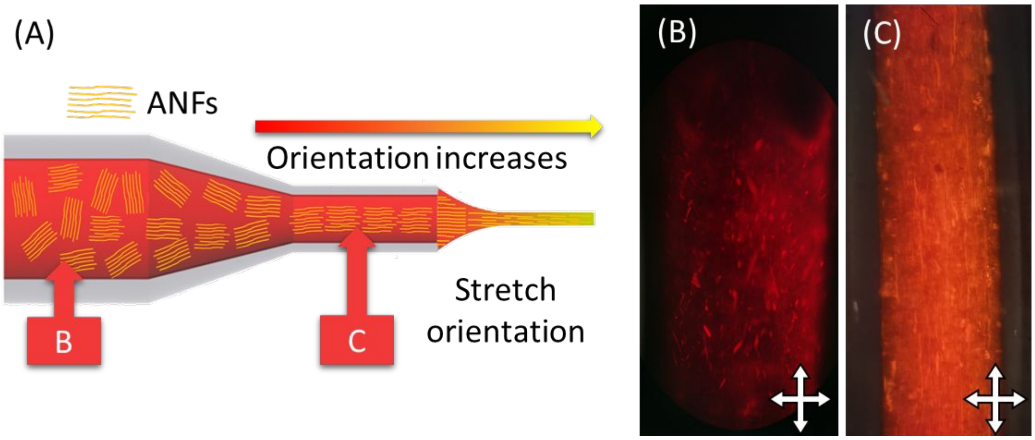

Figure S5. (a) Schematic illustration of the orientation of NKLC during the extrusion process. (b) The POM image of NKLC extruding at low speed (< 1.0 cm/s) in a thick pipe (with a diameter of 5.0 mm). (c) The POM image of NKLC extruding at high speed (15.0 cm/s) in a thin tube (with a diameter of 1.0 mm).

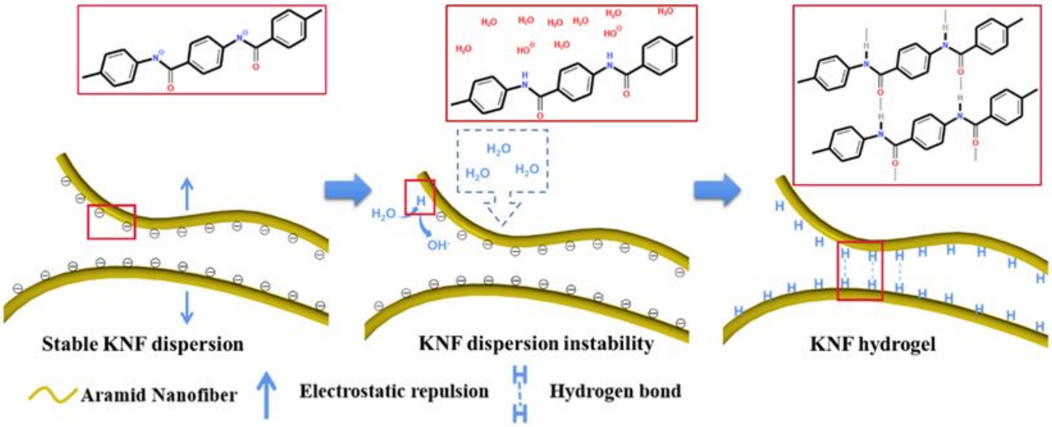

Figure S6. Schematic diagram of the sol-gel transition process to obtain the NKLC gel fibers.

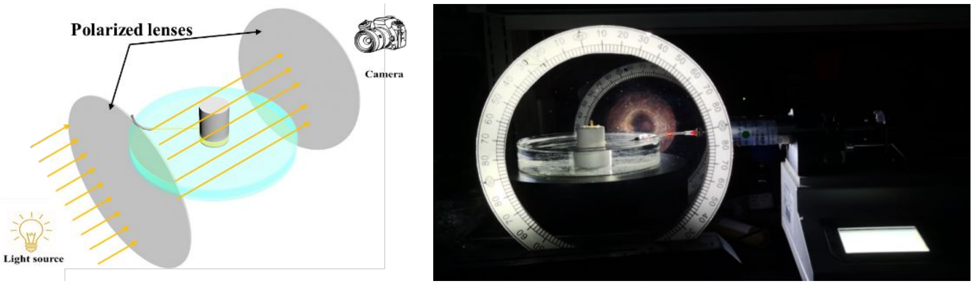

Figure S7. Schematic diagram and photograph of in-situ orientation detection equipment during liquid crystal spinning process.

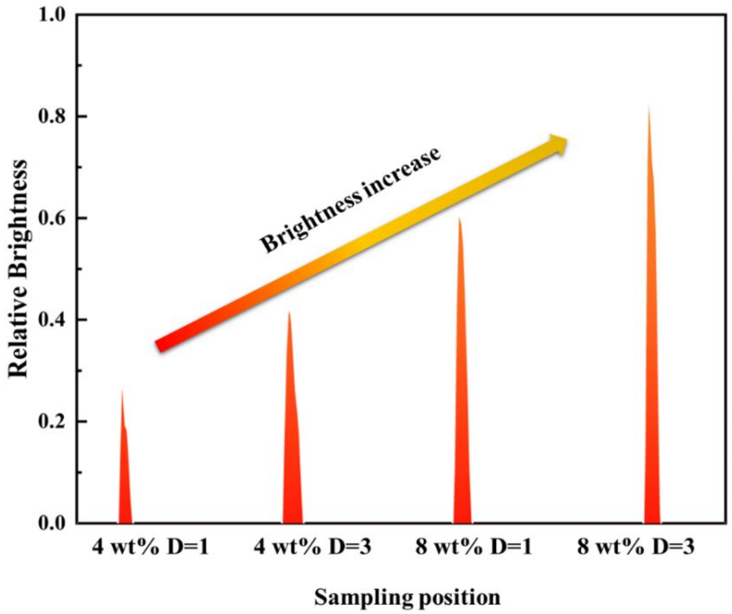

Figure S8. The relative brightness of the gel fibers was prepared with different Kevlar nanofiber concentrations and different draft ratios.

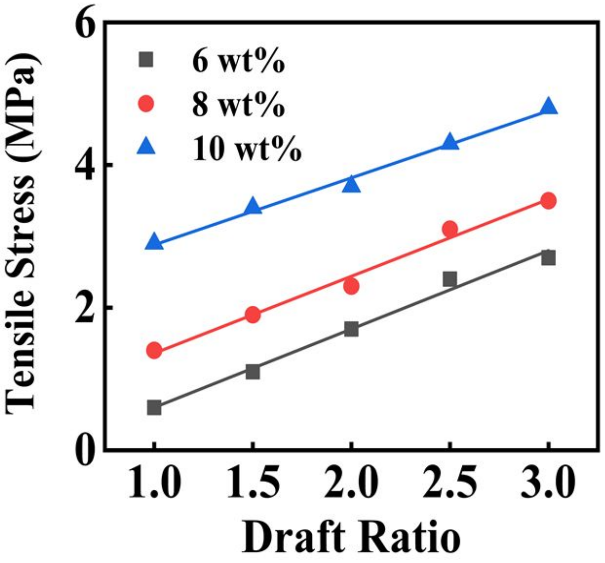

Figure S9. The mechanical tensile strength of the hydrogel fibers was prepared with different Kevlar nanofiber concentrations as a function of the draft ratio.

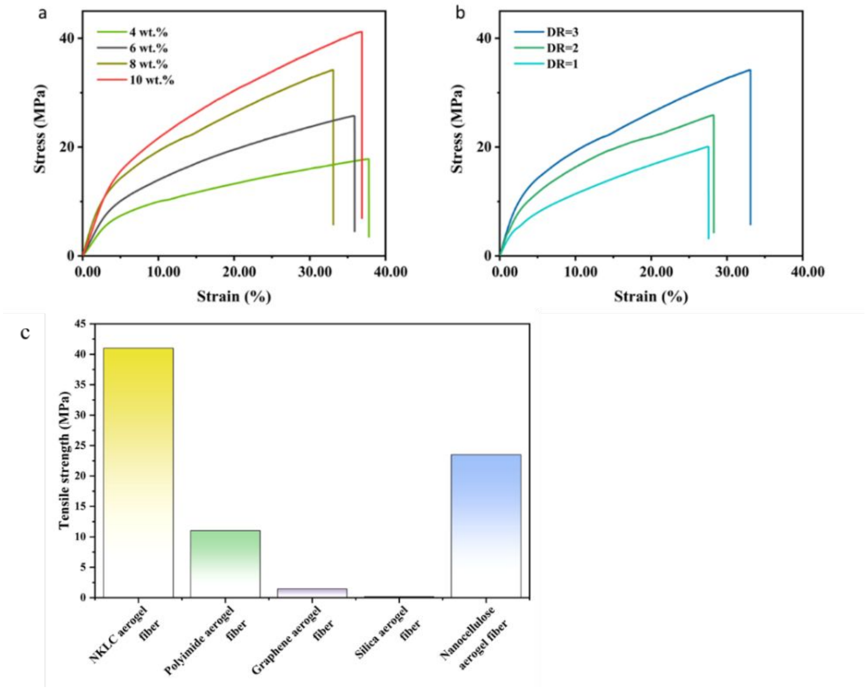

Figure S10. (a) The tensile stress-strain curves of the aerogel fiber prepared with different Kevlar nanofiber concentrations. (b) The tensile stress-strain curves of the NKLC aerogel fiber with different draft ratios. (c) The comparison of tensile strength of NKLC aerogel fibers with previously reported aerogel fibers[1-4].

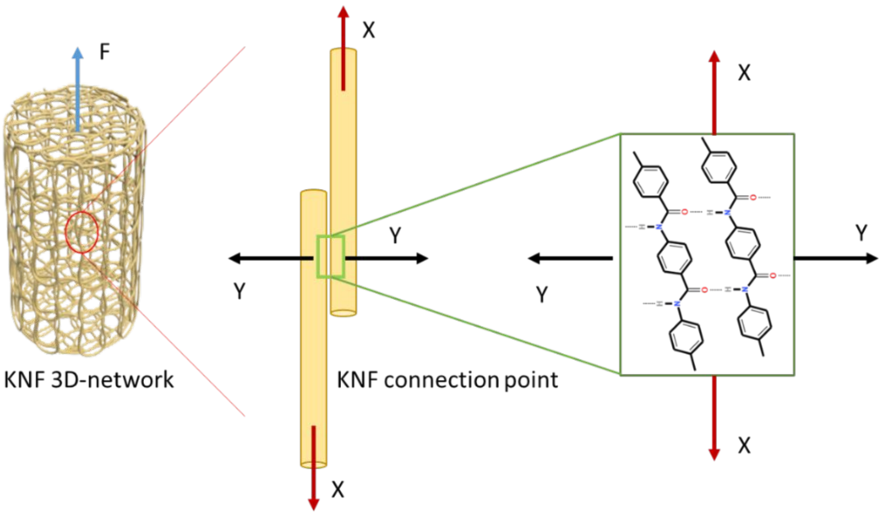

Figure S11. Schematic illustrating why both tensile strength and elongation at break increase as the orientation of the fiber increases.

The internal structure of NKLC aerogel fibers is a three-dimensional network structure. The Kevlar nanofibers are linked by hydrogen bonds and π-π stacking. When subjected to external force, the force directions of the connection points in the three-dimensional networks are different, which can be simplified into two directions, i.e., the drafting force along the molecular chain direction (X-direction) and the other direction (Y-direction). When external force is applied on the

aerogel fiber, the connection point subjected to the force in the Y-direction is broken first. When the nanofibers are stretched in the Y-direction, the nanofibers are broken when the distance between them is relatively small. When the X-direction is stretched, the molecular chains bound by the dense hydrogen bonds require a larger moving distance. Therefore, the tensile strength and elongation at break in the X direction are both higher than those in the Y-direction. As the orientation of the fibers increases, the number of nanofibers aligned along with the X-direction increases, resulting in simultaneous increases in the breaking strength and elongation at the break.

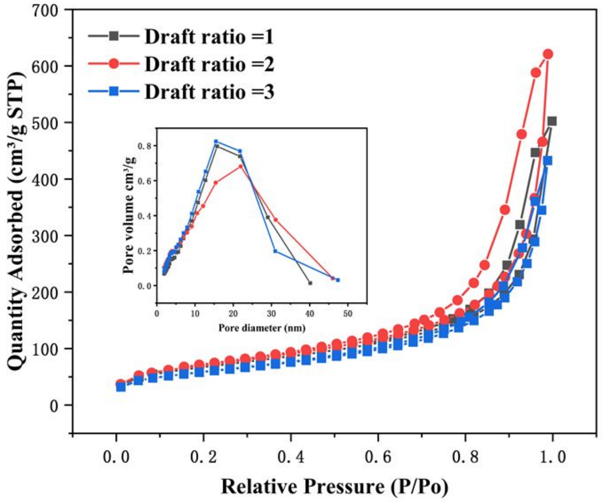

Figure S12. Nitrogen absorption-desorption isotherms of the NKLC aerogel fiber were prepared with different draft ratios. The inset is the corresponding pore volume. According to the BET method, the calculated specific surface area is 233 m^2/g, 245 m^2/g, and 204 m^2/g, for the NKLC aerogel fibers prepared with a draft ratio of 1, 2, and 3, respectively.

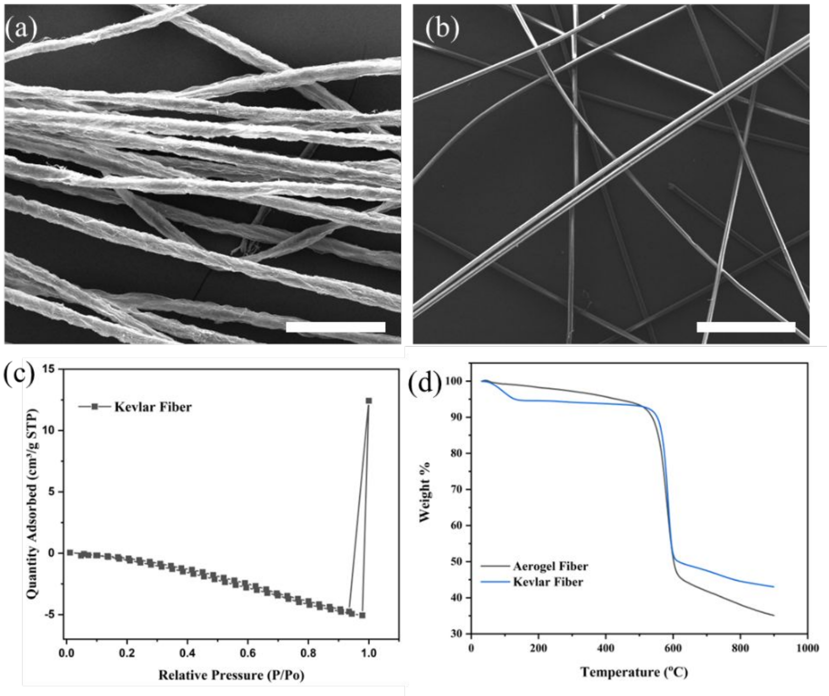

Figure S13. (a) SEM image of NKLC aerogel fiber with a scale bar of 100 μm. (b) SEM image of Kevlar fiber with a scale bar of 500 μm. (c) Nitrogen absorption-desorption isotherms of the Kevlar fiber. (d) TG curves of the Kevlar fiber and the aerogel fiber

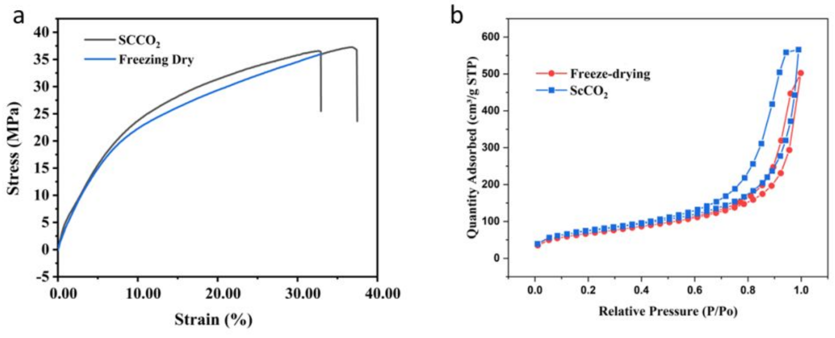

Figure S14. (a) The tensile stress-strain curves of the NKLC aerogel fibers prepared with $ScCO_2$ dying and freeze-dying. (b) Nitrogen absorption-desorption isotherms of the fibers prepared with $ScCO_2$ dying and freeze-dying. According to the BET method, the calculated specific surface area is 260 m^2/g and 233 m^2/g for the fibers prepared with $ScCO_2$ dying and freeze-dying, respectively.

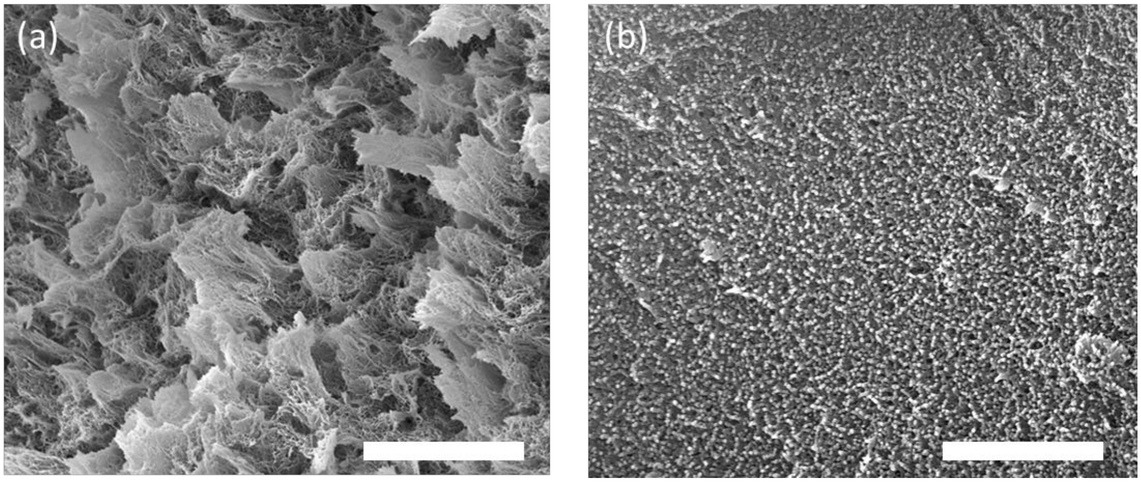

Figure S15. (a) Cross-section SEM image of DR1 aerogel fiber with a scale bar of 3 μm; (b) Cross-section SEM image of DR3 aerogel fiber with a scale bar of 3 μm.

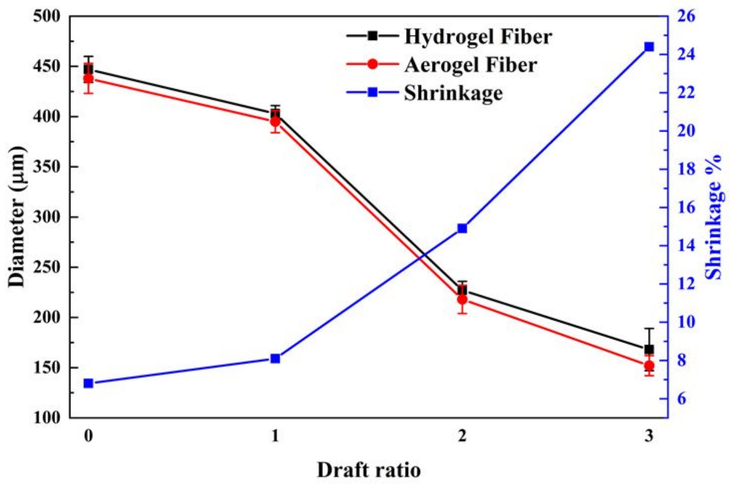

Figure S16. The diameter of the NKLC gel fibers and the corresponding aerogel fibers are prepared with different draft ratios and the drying shrinkage rate changes with different draft ratios.

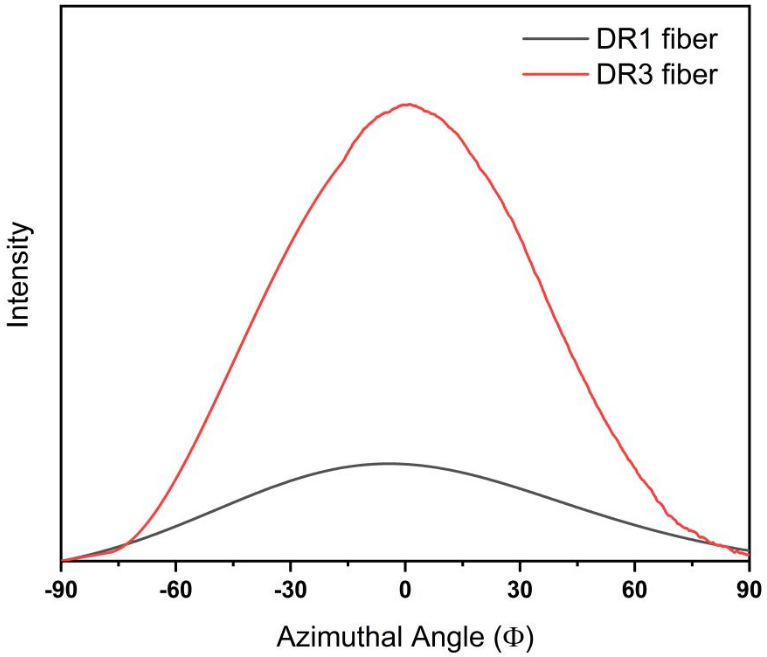

Figure S17. WAXS scanning intensity-azimuthal angle curve of different fibers.

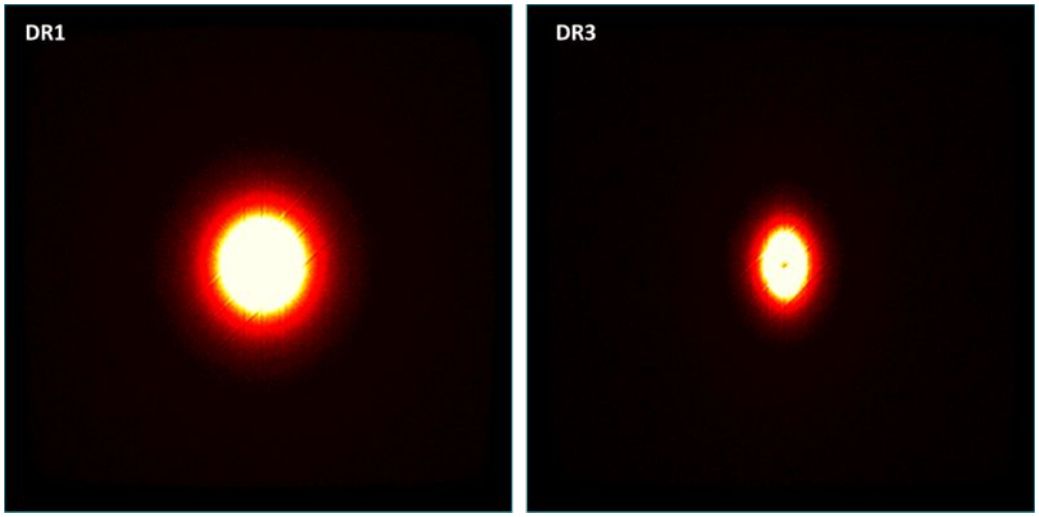

Figure S18. SAXS patterns of the DR1 and DR3 aerogel fibers.

.

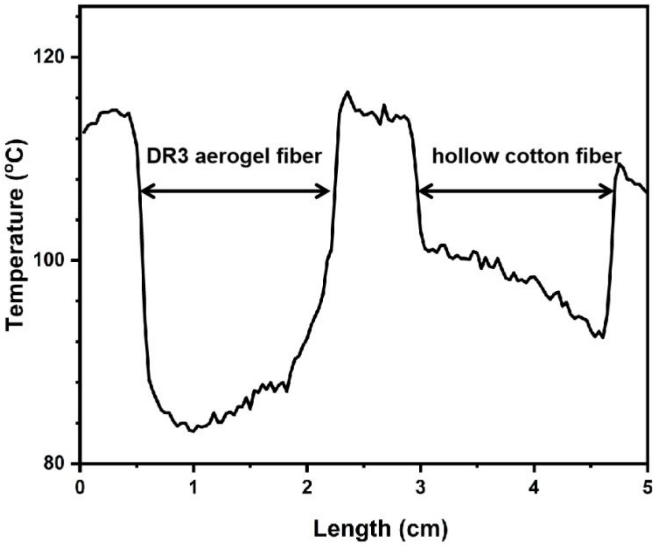

Figure S19. The temperature distribution profile was collected for the DR3 aerogel fiber mat and hollow cotton fiber mat from the high-temperature insulation test.

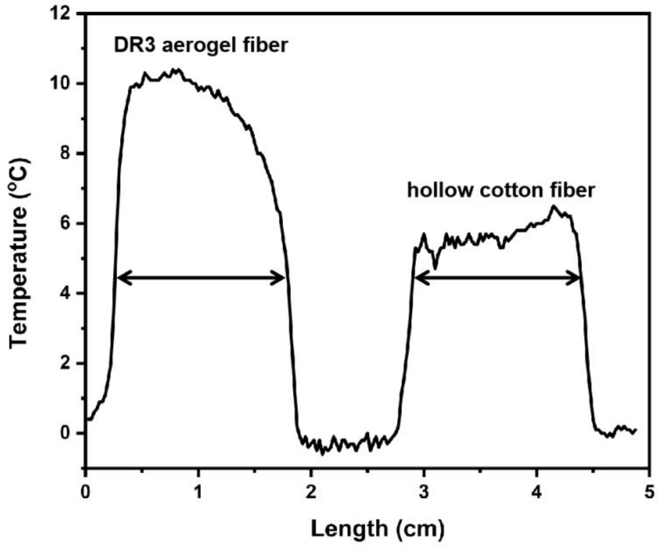

Figure S20. The temperature distribution profile was collected for the DR3 aerogel fiber mat and hollow cotton fiber mat from the low-temperature insulation test.

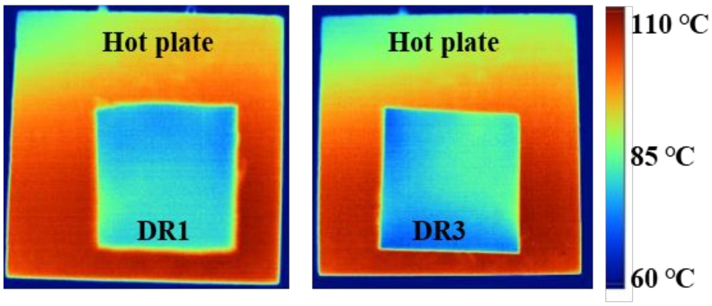

Figure S21. Infrared photos of the DR1 aerogel fiber and DR3 aerogel fiber on a hot plate.

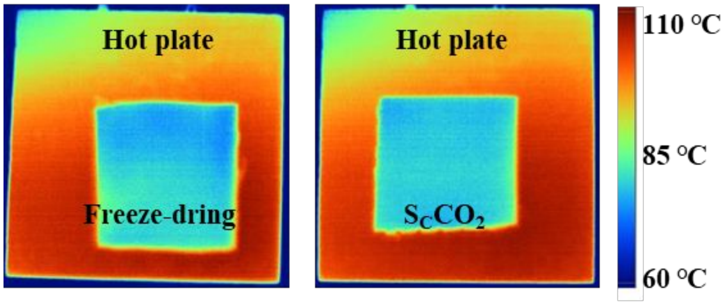

Figure S22. Infrared photos of the freeze-dried NKLC aerogel fiber and $ScCO_2$ dried NKLC aerogel fiber on a hot plate.

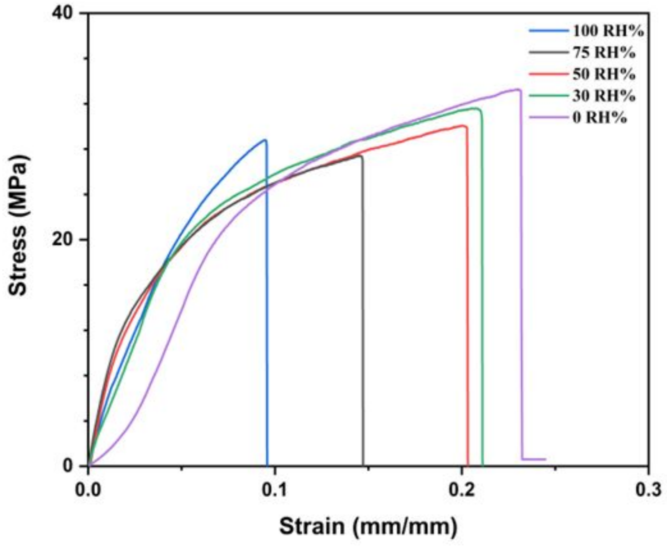

Figure S23. Stress-strain curves of the NKLC aerogel fibers under different humidity conditions.

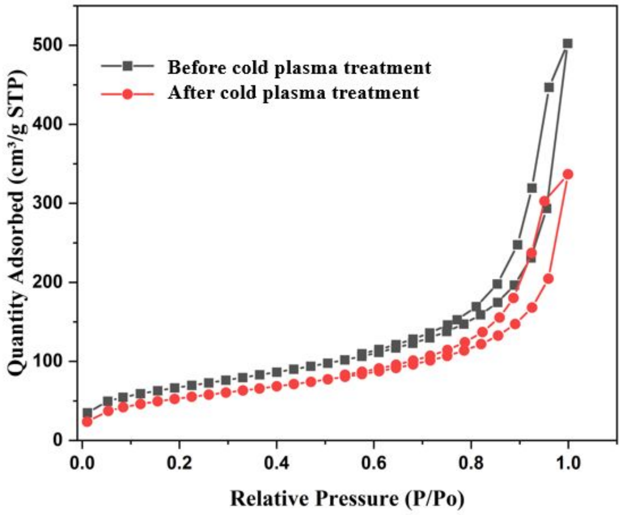

Figure S24. Nitrogen absorption-desorption isotherms of the NKLC aerogel fiber before and after cold plasma treatment. According to the BET method, the calculated specific surface area is 233 m^2/g and 192 m^2/g for the NKLC aerogel fibers before and after cold plasma treatment, respectively.

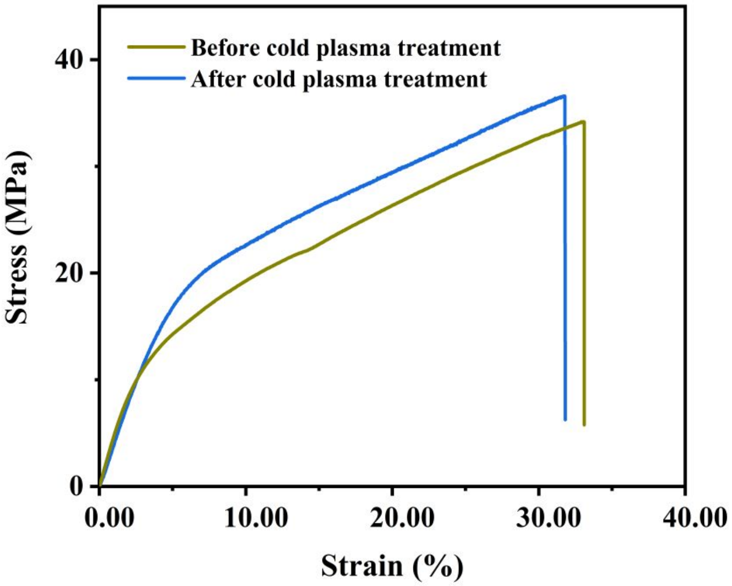

Figure S25. The tensile stress-strain curves of NKLC aerogel fiber before and after cold plasma treatment.

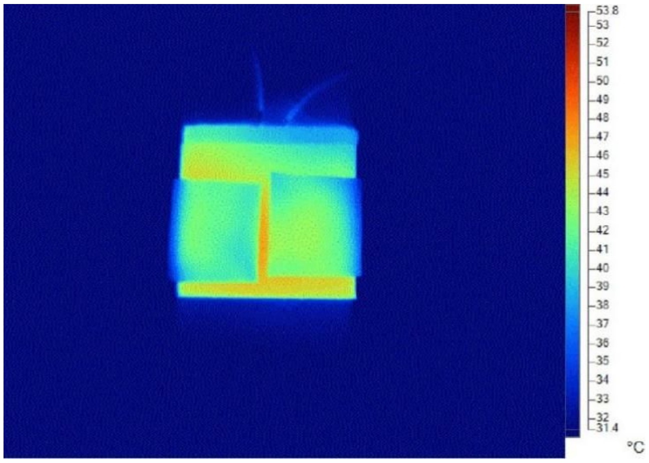

Figure S26. The infrared photo of the NKLC aerogel fiber textiles before (right) and after (left) cold plasma treatment on a hot plate.

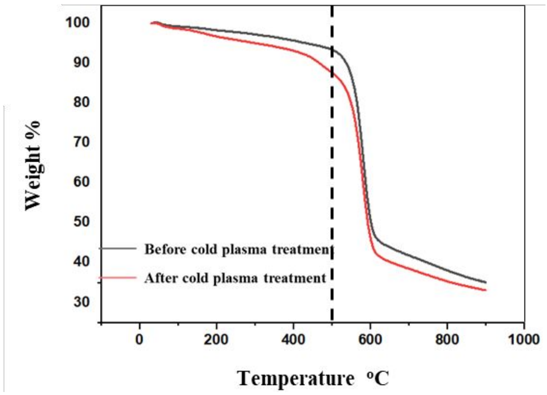

Figure S27. The TG curves of NKLC aerogel fiber textiles before and after cold plasma treatment.

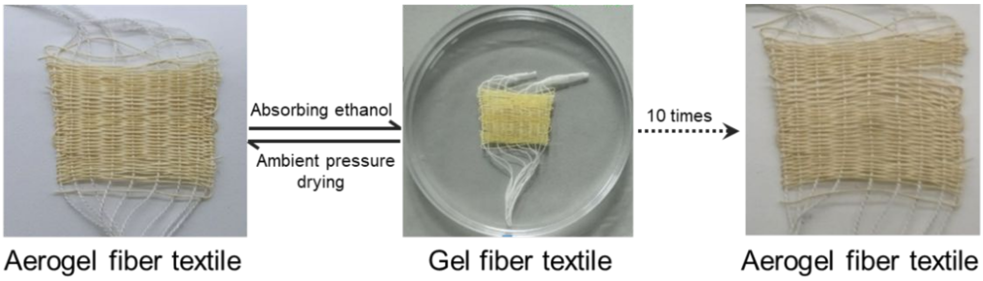

Figure S28. The textile was woven with superhydrophobic NKLC aerogel fiber transferred between aerogel-gel cyclically 10 times with ethanol absorbing and ambient pressure drying.

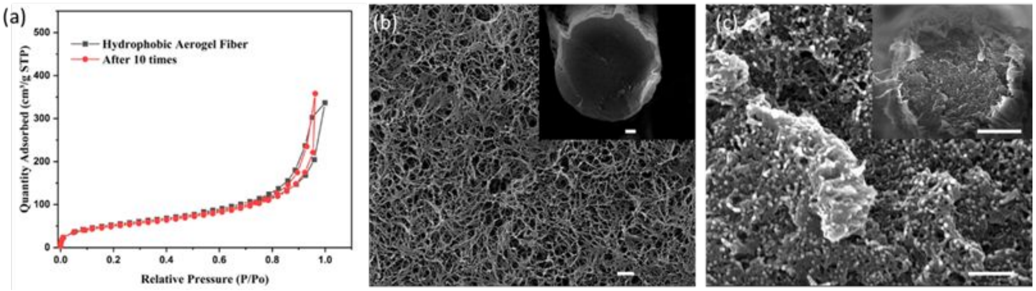

Figure S29. (a) Nitrogen absorption-desorption isotherms of the NKLC aerogel fiber before and after 10 aerogel-gel cycles. (b) Cross-section SEM image of DR1 aerogel fiber after 10 aerogel-gel cycles with a scale bar of 500 nm. The inset is its SEM image under low magnification with a scale bar of 10 μm. (c) Cross-section SEM image of DR3 aerogel fiber after 10 aerogel-gel cycles with a scale bar of 500 nm. The inset is its SEM image under low magnification with a scale bar of 10 μm.

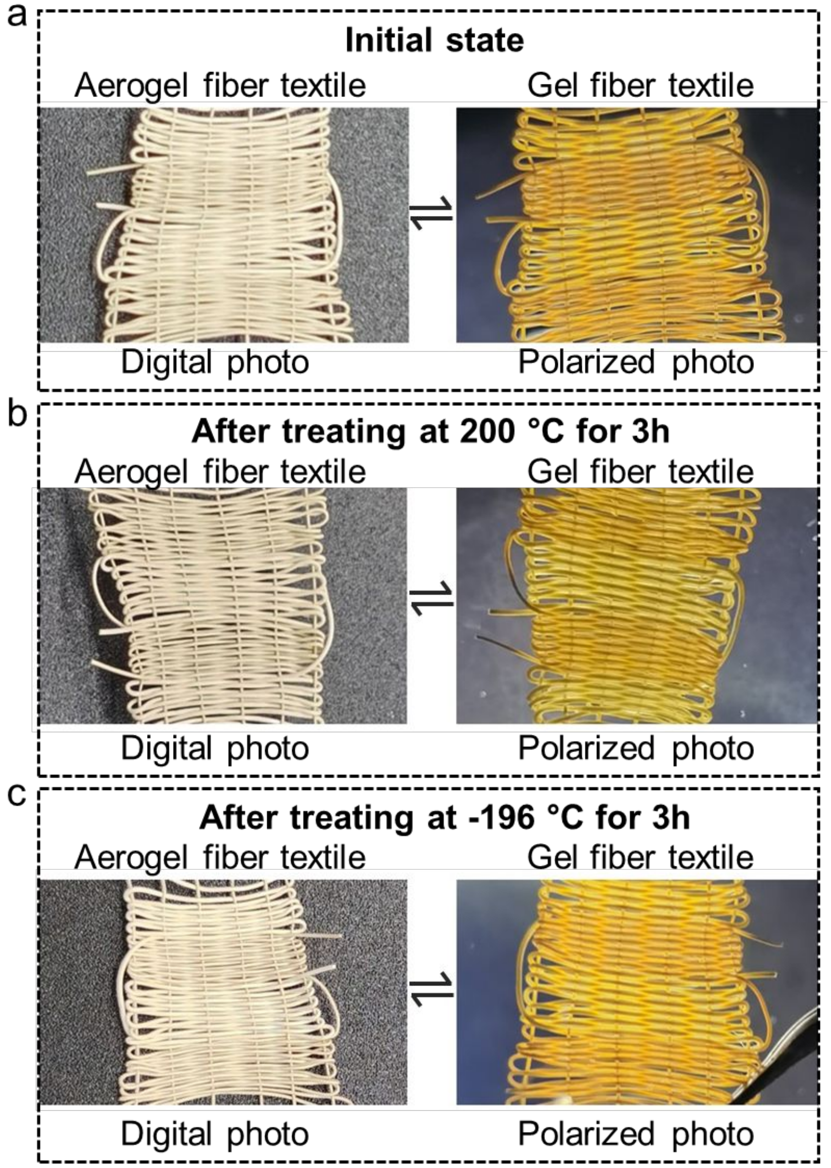

Figure S30. (a) Digital photo of the aerogel fiber textile is woven with DR1 and DR3 aerogel fibers (left) and a polarized optical photo of its corresponding gel fiber textile (right). (b) Digital photo of the aerogel fiber textile after treating at 200 ˚C for 3 h (left) and polarized optical photo of its

corresponding gel fiber textile (right). (c) Digital photo of the aerogel fiber textile after treating at -196 ˚C for 3 h (left) and polarized optical photo of its corresponding gel fiber textile (right).

Table S1. Contact angle to water of the aerogel fibers prepared with a different hydrophobic agent during cold plasma treatment.

hydrophobic agent	contact angle
dimethoxydimethylsilane	110.1 °
hexamethyldisiloxane	106.6 °
dichloromethane	106.1 °
1,2-dichloroethane	110.9 °
n-butyl bromide	114.0 °
Octamethylcyclotetrasiloxane (D4)	154.0 °

Table S2. Contact angle to water of the aerogel fibers prepared with different Kevlar nanofiber concentrations and different draft ratios (i.e., orientation degrees).

The concentration of Kevlar nanofiber	4 wt%	6 wt%	8 wt%	10 wt%
Draft ratio=1	152 °±3 °	150 °±2 °	154 °±3 °	149 °±4 °
Draft ratio=2	148 °±2 °	151 °±3 °	158 °±2 °	151 °±4 °

Draft ratio=3	150°±4°	151°±2°	155°±3°	157°±2°

References

(1) Li, X.; Dong, G.; Liu, Z.; Zhang, X. Polyimide Aerogel Fibers with Superior Flame Resistance, Strength, Hydrophobicity, and Flexibility Made via a Universal Sol-Gel Confined Transition Strategy. *ACS Nano* 2021, *15* (3), 4759-4768.

(2) Li, G.; Hong, G.; Dong, D.; Song, W.; Zhang, X. Multiresponsive Graphene-Aerogel-Directed Phase-Change Smart Fibers. *Adv Mater* 2018, *30* (30), e1801754.

(3) Du, Y.; Zhang, X.; Wang, J.; Liu, Z.; Zhang, K.; Ji, X.; You, Y.; Zhang, X. Reaction-Spun Transparent Silica Aerogel Fibers. *ACS Nano* 2020, *14* (9), 11919-11928.

(4) Zhou, J.; Hsieh, Y. L. Nanocellulose aerogel-based porous coaxial fibers for thermal insulation. *Nano Energy* 2020, *68*.
